# Alpha-helicoidal HEAT-like Repeat Proteins (αRep) Selected as Interactors of HIV-1 Nucleocapsid Negatively Interfere with Viral Genome Packaging and Virus Maturation

**DOI:** 10.1038/s41598-017-16451-w

**Published:** 2017-11-27

**Authors:** Sudarat Hadpech, Sawitree Nangola, Koollawat Chupradit, Kanda Fanhchaksai, Wilhelm Furnon, Agathe Urvoas, Marie Valerio-Lepiniec, Philippe Minard, Pierre Boulanger, Saw-See Hong, Chatchai Tayapiwatana

**Affiliations:** 1 Division of Clinical Immunology, Department of Medical Technology, Faculty of Associated Medical Sciences, Chiang Mai University, Chiang Mai, 50200 Thailand; 2 Center of Biomolecular Therapy and Diagnostic, Faculty of Associated Medical Sciences, Chiang Mai University, Chiang Mai, 50200 Thailand; 3 Faculty of Pharmaceutical Science, Burapha University, Muang District, Chonburi Province, 20131 Thailand; 4 University Lyon 1, UMR754-INRA-EPHE, Viral Infections and Comparative Pathology, 50, Avenue Tony Garnier, 69366 Lyon Cedex 07, France; 5 Division of Clinical Immunology and Transfusion Sciences, School of Allied Health Sciences, University of Phayao, Phayao, 56000 Thailand; 6 Institute for Integrative Biology of the Cell (I2BC), CEA, CNRS, Université Paris-Sud, Université Paris-Saclay, 91198 Gif-sur-Yvette cedex, France; 7 Institut National de la Santé et de la Recherche Médicale, 101, rue de Tolbiac, 75654 Paris Cedex 13, France

## Abstract

A new generation of artificial proteins, derived from alpha-helicoidal HEAT-like repeat protein scaffolds (αRep), was previously characterized as an effective source of intracellular interfering proteins. In this work, a phage-displayed library of αRep was screened on a region of HIV-1 Gag polyprotein encompassing the C-terminal domain of the capsid, the SP1 linker and the nucleocapsid. This region is known to be essential for the late steps of HIV-1 life cycle, Gag oligomerization, viral genome packaging and the last cleavage step of Gag, leading to mature, infectious virions. Two strong αRep binders were isolated from the screen, αRep4E3 (32 kDa; 7 internal repeats) and αRep9A8 (28 kDa; 6 internal repeats). Their antiviral activity against HIV-1 was evaluated in VLP-producer cells and in human SupT1 cells challenged with HIV-1. Both αRep4E3 and αRep9A8 showed a modest but significant antiviral effects in all bioassays and cell systems tested. They did not prevent the proviral integration reaction, but negatively interfered with late steps of the HIV-1 life cycle: αRep4E3 blocked the viral genome packaging, whereas αRep9A8 altered both virus maturation and genome packaging. Interestingly, SupT1 cells stably expressing αRep9A8 acquired long-term resistance to HIV-1, implying that αRep proteins can act as antiviral restriction-like factors.

## Introduction

Although highly active antiretroviral therapy (HAART) has significantly reduced the morbidity and mortality associated with AIDS, curative therapy has been greatly impaired by the occurrence of drug resistant mutants and the persistence of virus in a latent form in reservoirs that resist current HAART^1–4^. The high mutation rate of the human immunodeficiency virus 1 (HIV-1) and the persistence of viruses in tissue sanctuaries impose constant efforts to develop new antiviral drugs and new strategies^5,6^. Alternative strategies include the design of genes coding for intracellular factors or interactors with antiviral activity, the genetic manipulation of hematopoietic progenitor stem cells^7^, and the inactivation of proviral DNA *in situ* by using zinc-finger nucleases (ZFNs), transcription activator-like effector nucleases (TALENS), or the clustered regularly interspaced short palindromic repeat/Cas9 (CRISPR) system^8^. A recent example of the design of novel antivirals based on HIV-1 interactors was given by LEDGINs^9,10^, allosteric inhibitors of integrase (IN) which block the interaction of IN with lens epithelium-derived growth factor (LEDGF) or p75^11^.

Among the anti-HIV therapies using intracellular protein interference, protein-based molecular scaffolds are considered as promising antivirals. Antibodies, their derivatives scFv and intrabodies, and single domain antibodies from *Camelidae* (or nanobodies) are the most commonly used scaffolds to bind protein targets. However, the proper folding, stability and biological activity of these molecules require inter- or intra-domain disulfide bond formation. This constitutes severe limitations to their development as intracellular antivirals, considering the reducing environment of the cytoplasm. In order to overcome these limitations, other disulfide-free, protein-based molecular scaffolds have been designed, such as artificial ankyrin-repeat proteins (Anks) and the ankyrin derivatives DARPINS^12–18^. Some of these scaffolds are already in preclinical studies for the treatment of cancer^19^, and others, like DARPINS or Anks, have been tested *in vitro* against HIV-1 infection, and have been found to act at the initial step of binding of the virus to its cell surface receptors^20^, or at post-entry steps^21,22^.

We have previously designed and characterized two intracellular inhibitors of HIV-1 replication, abbreviated 2LTRZFP and Ank^GAG^1D4, which are based on stable modular protein scaffolds. 2LTRZFP is a designed zinc finger protein (ZFP) which targets the integrase recognition sequence at the 2-LTR circle junctions, and blocks the integration of the HIV-1 cDNA into the host cell genome^23,24^. Ank^GAG^1D4 is an artificial ankyrin-repeat protein selected as a binder of the N-terminal domain of HIV-1 capsid protein (CA), which was capable of interfering negatively with viral assembly in HIV-1-infected SupT1 cells^21,22^. Interestingly, the combined expression of 2LTRZFP and Ank^GAG^1D4 molecules in HIV-1-infected cells resulted in a significant negative effect on the viral replication^25^.

Another type of molecular scaffold, named alpha-repeat proteins (αRep), were tested as potential antivirals against HIV-1 in the present work. The αRep proteins were derived from a natural family of modular proteins constituted of alpha-helical repeats, related to HEAT repeats, named after Huntingtin, the **e**longation factor 3 (EF3), the protein phosphatase 2**A** (PP2A), and the yeast kinase TOR1^26–29^. The association of several HEAT repeats forms alpha-solenoids of various lengths, which are naturally found in a number of cellular proteins involved in intracellular transport and protein-protein interaction^26,28^. The biophysical properties of αRep proteins are highly favourable to biological and medical applications: (i) αRep proteins are easily expressed in bacteria as soluble proteins, implying a properly folded protein; (ii) they are functional in reducing and oxidative environments due to their disulfide independent folding, and can be active inside living cells as well as in the extracellular milieu; (iii) they are thermostable, with Tm >70 °C; (iv) heat denaturation is reversible and αRep proteins refold properly when cooled to 25 °C; (v) aggregation of αRep proteins does not occur at high concentrations; (vi) their small size allows the recognition of epitopes of low accessibility, thus increasing the diversity of binders for one target^28,29^. All these biological properties of αRep proteins open a wide range of possibilities for their development as diagnostic and therapeutic tools, or for cellular imaging.

In this study, we selected αRep proteins which bind to a region of HIV-1 Pr55Gag polyprotein which encompasses the last 21 residues of the capsid (CA_21_), the SP1 linker and the nucleocapsid (NC) protein with its two zinc fingers. This region contained several elements identified as privileged targets for antiviral drugs. (i) SP1, the spacer peptide separating the capsid from the nucleocapsid domain, is a 14-residue long, alpha-helical domain which is critical for the self-assembly of Pr55Gag, and is the target of betulinic acid-derived assembly inhibitors^30–35^. (ii) The NC occupies a strategic position in the HIV-1 infectious cycle, as it possesses structural and catalytic properties, and also acts as a nucleic acid chaperone^36–40^. The multiple functions in which NC is involved^41–45^, as well as its ALIX-mediated role in the virus particle release^46,47^, justified the inclusion of the NC domain in our viral bait.

Two αRep molecules with a high binding activity, αRep4E3 and αRep9A8, were isolated from our screen. When the intracellular antiviral functions were assessed *in vitro*, both αRep proteins showed a modest but significant inhibitory effect on HIV-1 replication, although interestingly *via* different mechanisms. αRep4E3 interfered negatively with the viral genome packaging, whereas αRep9A8 mainly impaired the proteolytic processing of Gag, the step required for the transition of immature particles to infectious virions. Importantly, a SupT1 cell line stably expressing the αRep9A8 protein was found to acquire a long-term resistance to HIV-1 infection, with the production of noninfectious virus particles at background levels. These results might have significant implications in the elaboration of future strategies of anti-HIV-1 cell therapy, based on artificial restriction-like factors.

## Results

### Selection of αRep binders of the HIV-1 Gag C-terminal domain

**Figure 1:**
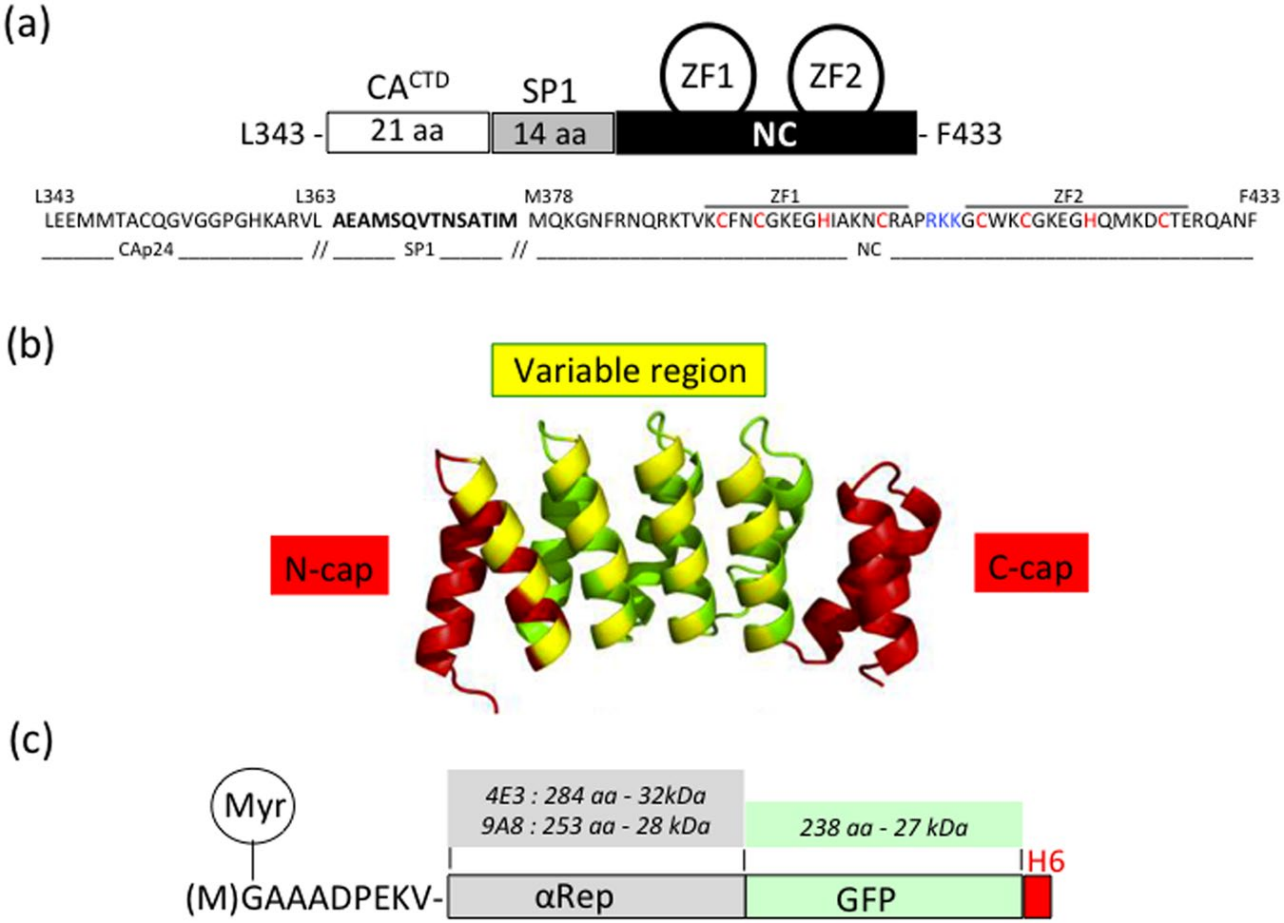
Schematic representation of the protein partners. (**a**) Schematic representation of the viral target, spanning residues L343 to F433 of HIV-1 Pr55Gag, with its amino acid sequence (HIV-1_LAI_ isolate) shown underneath. Amino acid residues of the SP1 domain are in bold; the cysteine and histidine residues of the zinc fingers (ZF1 and ZF2) responsible for the Zn coordinates are in red; the residues of the basic motif separating ZF1 and ZF2 are in blue. (**b**) Three-dimensional model of an αRep molecule. The constant regions N-cap and C-cap are shown in red, and the variable region, comprising of four internal alpha-repeats in this particular model (*n* = 4), is in yellow and green. (**c**) Schematic representation of an αRep-GFP fusion protein, with the N-terminus modified to carry a N-myristoylation signal (Myr), and a His-tag at the C-terminus. The respective number of amino acids (aa), and molecular weight of αRep4E3, αRep9A8 and GFP domains are indicated in kDa.

**Figure 2:**
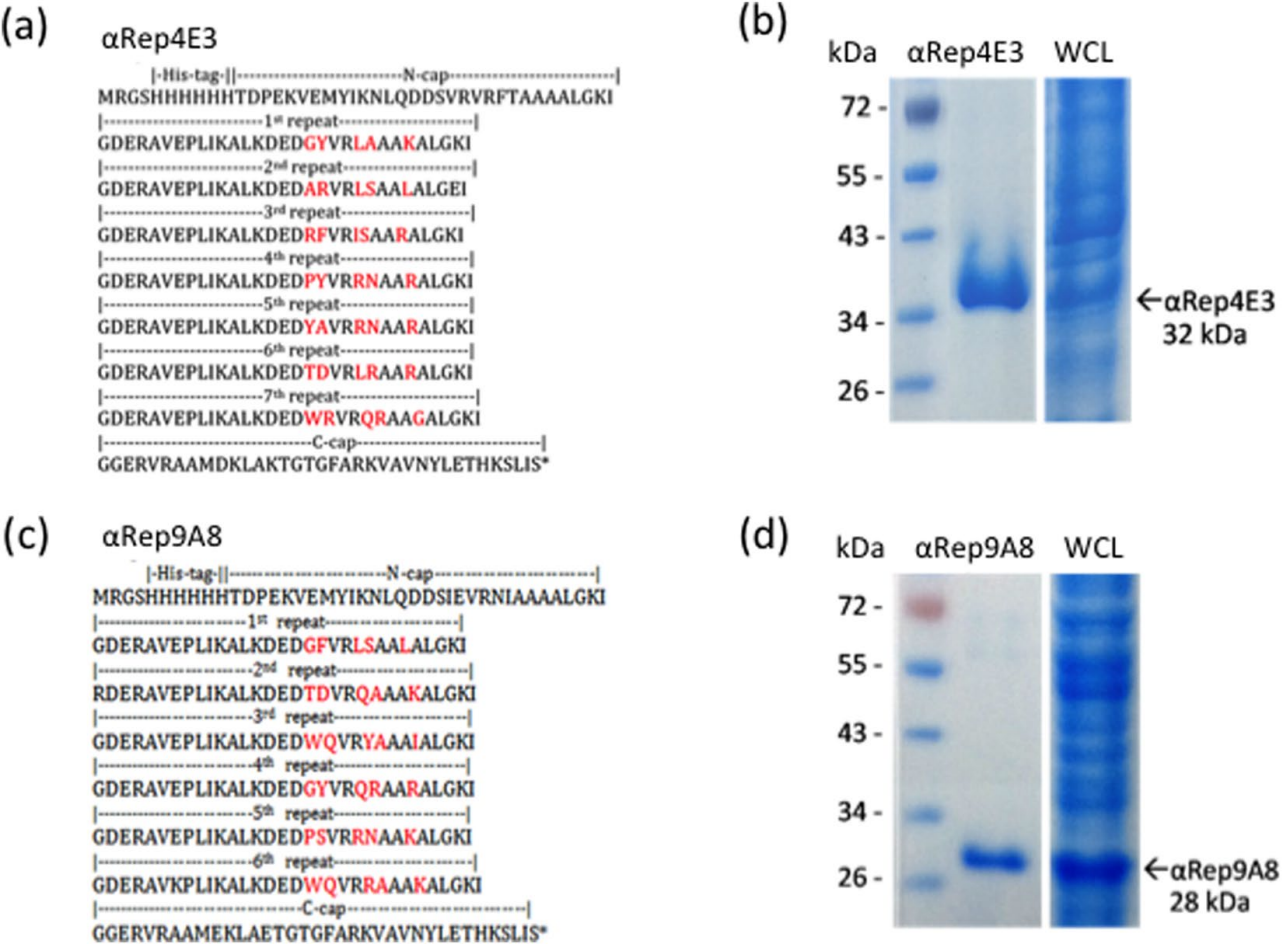
Biochemical characteristics of αRep4E3 and αRep9A8. (**a**,**c**) Amino acid sequence of αRep4E3 (**a**) and αRep9A8 (**c**) with the variable residues of the internal repeats shown in red, and constant residues in black. (**b,d**) SDS-PAGE and Coomassie blue staining of affinity-purified, His-tagged αRep proteins (middle lane); whole bacterial cell lysates (WCL) are shown on the right lane, and prestained molecular mass markers on the left lane.

Our viral bait, abbreviated CA_21_-SP1-NC, spanned from residue L343 to F433 of the Pr55Gag sequence (Fig. 1a). GST-fused CA_21_-SP1-NC was used for the selection of specific binders from a phage-displayed, random library of artificial αRep proteins generated by combinatorial methods^26,27^. After three rounds of phage biopanning, 30 individual clones out of an original library of ~1.7 × 10^9^ independent bacteriophages, were randomly picked and analyzed by ELISA. Eighteen clones were found to react positively with the immobilized bait, and six of them, αRep4D4, -4E3, -4F2, 5A12, -5F1 and -9A8, showed a significantly higher signal compared to the twelve other clones. The genes of these six αRep were sub-cloned into the pQE-31 expression vector for the production of His-tagged αRep proteins in *E. coli*. After purification by affinity chromatography on Ni^2+^-column, the six selected αRep proteins were analyzed for their binding activity to GST-CA_21_-SP1-NC *in vitro*, using an indirect ELISA method. Out of the six clones, αRep4E3 and αRep9A8 showed the highest binding reaction with the viral target (not shown), and were therefore kept for further characterization. DNA sequencing showed that αRep4E3 and αRep9A8 had in common a His-tag at their N-terminus, the flanking N-terminal cap (N-cap) and C-terminal cap (C-cap) sequences, and the constant regions of the internal repeat modules (Fig. 1b). αRep4E3 had seven internal repeat modules, 284 amino acids (including N-cap and C-cap) and a molecular mass of 32 kDa, while αRep9A8 had six internal repeats, 253 amino acids and a molecular mass of 28 kDa (Fig. 2).

### Protein pull-down assays

**Figure 3:**
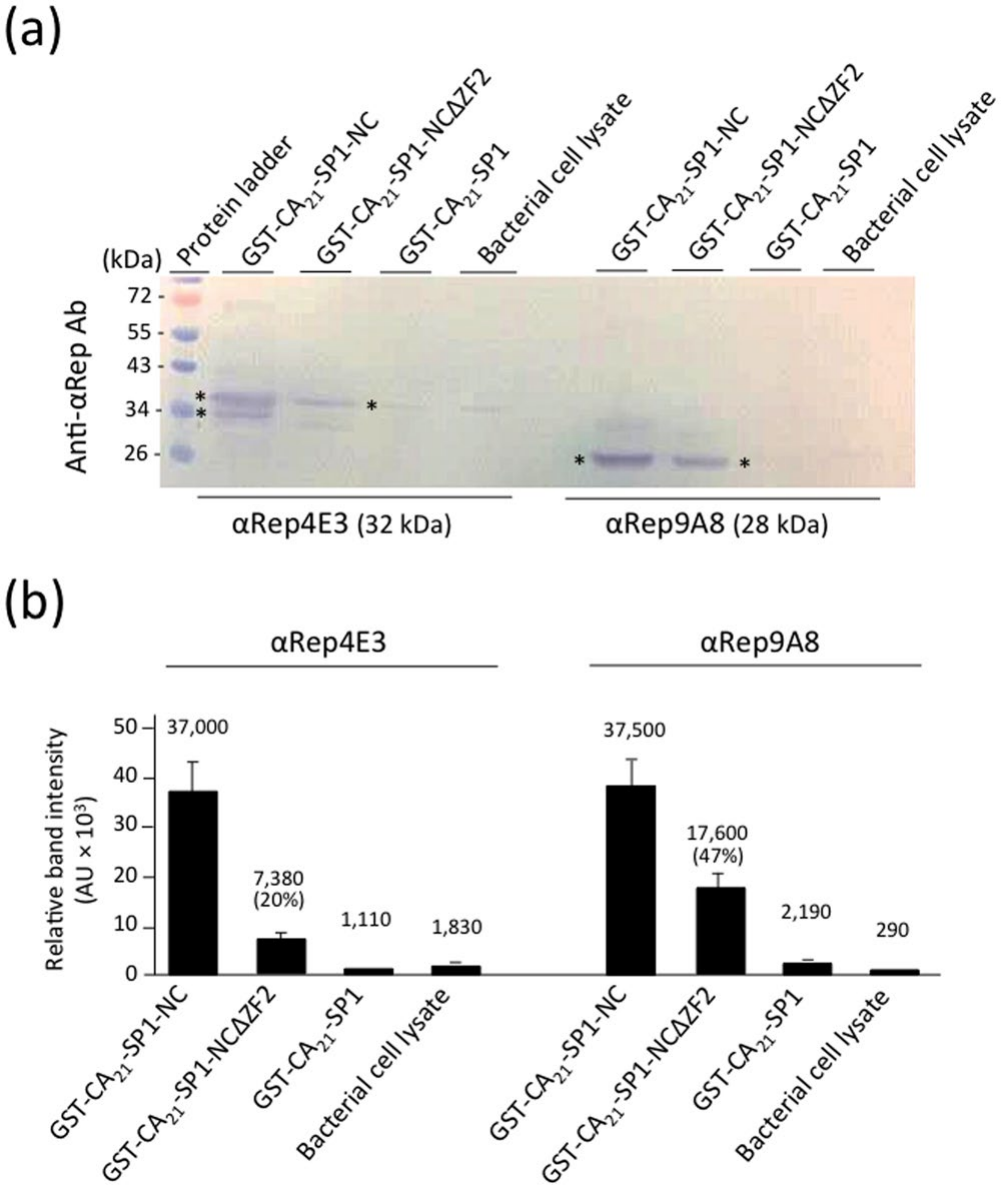
Protein pull-down analysis of αRep binding to the Gag target. Aliquots of bacterial cell lysates containing the full-length target GST-CA_21_-SP1-NC, or the carboxy-truncated mutants GST-CA_21_-SP1-NC∆ZF2 or GST-CA_21_-SP1 were incubated with purified αRep4E3 or αRep9A8, and protein complexes, isolated on glutathione-coated agarose beads, were analyzed by SDS-PAGE and Western blotting. **(a)** Blot reacted with rabbit anti-αRep antibody, followed by alkaline phosphatase-conjugated goat anti-rabbit IgG antibody. Asterisks indicate the immunoreactive bands of αRep4E3 and αRep9A8 proteins. Note the doublet band of αRep4E3 protein, suggesting some degree of proteolytic cleavage. **(b)** Bar graph of the scanning of αRep4E3 and αRep9A8 proteins bound to the different viral targets. The level of αRep binding to the full-length target GST-CA_21_-SP1-NC was attributed the 100% value.

The interaction of αRep4E3 and αRep9A8 with the viral target and the mapping of their binding sites on CA_21_-SP1-NC was investigated using protein pull-down assays. Purified His-tagged αRep proteins were incubated with the full-length viral target CA_21_-SP1-NC, or its carboxy-terminal deletants, GST-CA_21_-SP1-NCΔZF2 (lacking the downstream ZF2), and GST-CA_21_-SP1 (lacking the whole NC domain; see Supplementary Table S1). Protein complexes were isolated on glutathione-coated agarose beads, and analyzed by SDS-PAGE and Western blotting. Both αRep4E3 and αRep9A8 bound equally well to CA_21_-SP1-NC, but no binding was detectable with the NC-deleted target (Fig. 3a). Deletion of ZF2 significantly decreased the binding of both αRep, suggesting an overlap of their binding sites on the Gag target. However, differences were observed between the two binding patterns. The amounts of αRep complexed with GST-CA_21_-SP1-NCΔZF2 were 5-fold lower for αRep4E3 and 2-fold lower for αRep9A8, compared to their complex with full-length GST-CA_21_-SP1-NC (Fig. 3b). This suggested that the major binding determinants of αRep4E3 mapped to ZF2, with minor determinants in ZF1, whereas for αRep9A8, the binding determinants were distributed between the ZF1 and the ZF2 domains.

### Effects of αRep4E3 and αRep9A8 proteins on HIV-1 VLP assembly and extracellular release: quantitative aspects

The effects of the two αRep on HIV-1 assembly were explored using a baculovirus-insect cell system of production of HIV-1 virus-like particles (VLPs). In this system, Sf9 cells were infected with AcMNPV^*gag*^, a recombinant vector derived from the baculovirus AcMNPV (*Autographa californica* Multiple Nuclear Polyhedrosis Virus). AcMNPV^*gag*^ expresses the full-length, wild-type, N-myristoylated HIV-1 Gag polyprotein in Sf9 cells, resulting in the assembly and extracellular release of membrane-enveloped VLPs at high yields. The high number of particles produced per cell in this heterologous model system of VLP assembly and egress, allowed quantitative analyses of morphologically normal VLPs compared to aberrant particles^31,33,48–51^.

#### Expression of αRep in insect cells

The αRep4E3 and αRep9A8 genes were fused to the *GFP* gene and to the coding sequence of a six-histidine tag at the C-terminus (Fig. 1c). The coding sequence of an N-myristoylation signal^22,52^ was inserted to the 5′-end of the αRep-GFP genes (Fig. 1c). The resulting genetic constructs were transfected into Sf9 cells, and the two Sf9-derived cell lines thus obtained, Sf/(Myr+)αRep4E3-GFP and Sf/(Myr+)αRep9A8-GFP, stably expressed N-myristoylated (Myr+)αRep4E3-GFP and (Myr+)αRep9A8-GFP proteins under the control of the baculoviral OpIE2 promoter (see Supplementary Fig. S1). The aim of the N-myristoylation of αRep4E3-GFP and αRep9A8-GFP was to compensate for the difference in cell content between the N-myristoylated Pr55Gag polyprotein, expressed at high levels under the control of the strong polyhedrin promoter^31,33,48–51^, and αRep proteins, expressed at lower levels under the control of the weaker OpIE2 promoter. Addressing (Myr+)αRep proteins to the plasma membrane was meant to promote the interaction between Pr55Gag and αRep proteins at the assembly sites of membrane-enveloped VLPs.

#### Quantitative effect of (Myr+)αRep4E3 and (Myr+)αRep9A8 on VLP assembly

Control, nonmodified Sf9 cells, Sf/(Myr+)αRep4E3-GFP and Sf/(Myr+)αRep9A8-GFP cells were infected with AcMNPV^*gag*^, and the amounts of VLPs assembled and released into the culture medium were estimated by SDS-PAGE and Western blot analysis of pelletable, extracellular Pr55Gag^31–33^. The intensity of the Pr55Gag band on immunoblots did not radically differ between the three samples (data not shown). A quantitative, luciferase-based assay of extracellular VLPs was then performed. The principle of this assay resides in the co-packaging of Vpr (and Vpr-fused proteins) with Pr55Gag into membrane-enveloped VLPs banding at a density of 1.18. This co-packaging is mediated by the specific interaction between the Gag p6 domain and Vpr^53,54^. The co-expression of the Luciferase-Vpr fusion protein together with Pr55Gag by co-infection of Sf9 with the recombinant vectors AcMNPV^*luc-vpr*^ and AcMNPV^*gag*^ showed that the activity of luciferase co-incorporated with Pr55Gag into VLPs directly reflected the amounts of VLPs released in the extracellular medium^33^.

Control Sf9 cells, Sf/(Myr+)αRep4E3-GFP and Sf/(Myr+)αRep9A8-GFP cells were co-infected with AcMNPV^*gag*^ and AcMNPV^*luc-vpr*^, and harvested at 48 hrs pi. Cell lysates and gradient fractions were assayed for luciferase activity, and the results of VLP-associated luciferase activity were normalized to the cellular luciferase levels^33^. A peak of luciferase activity was detected at the density of VLPs in all three culture media and at similar levels (see Supplementary Fig. S2), which implied minor differences in the amounts of VLPs recovered from the different cell lines. This suggested that (Myr+)αRep4E3 and (Myr+)αRep9A8 were not able to completely block the assembly and extracellular release of VLPs from AcMNPV^*gag*^-infected insect cells.

### Effects of αRep4E3 and αRep9A8 on HIV-1 VLP assembly and extracellular release: qualitative aspects

**Figure 4:**
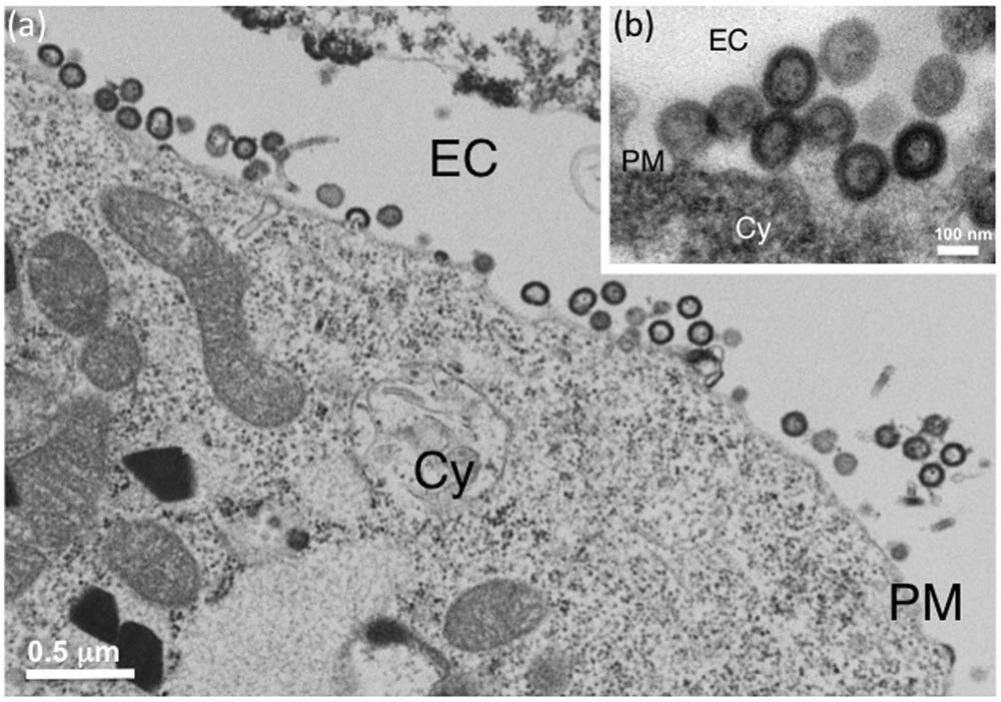
Electron microscopy (EM) of VLP assembly and budding from control insect cells. Control Sf9 cells were infected with recombinant baculovirus AcMNPV^*gag*^ expressing the HIV-1 Pr55Gag polyprotein, harvested at 48hrs post-infection, and processed for EM observation. Electron micrographs of ultrathin sections of cell pellets showing VLPs budding at their surface are presented at low (**a**), and high magnification (inset, **b**). A*bbreviations*: EC, extracellular space; PM, plasma membrane; Cy, cytoplasm.

**Figure 5:**
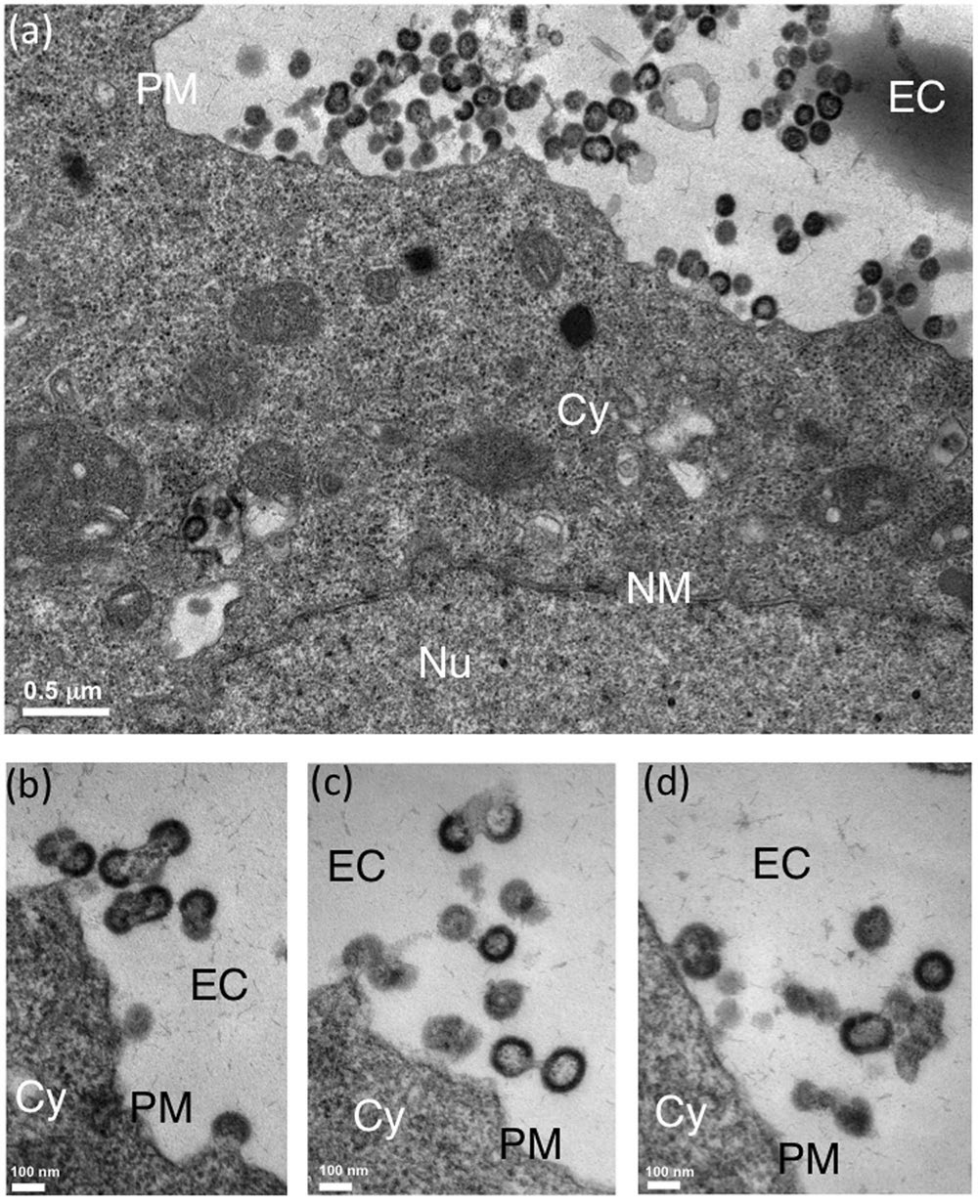
EM analysis of VLP assembly in αRep4E3-expressing insect cells. Sf/(Myr+)αRep4E3-GFP cells were infected with AcMNPV^*gag*^, harvested at 48hrs post-infection and processed for EM. Electron micrographs of ultrathin sections of cell pellets are shown at low (**a**), and high magnifications (**b-d**). EC, extracellular space; PM, plasma membrane; Cy, cytoplasm; Nu, nucleoplasm; NM, nuclear membrane.

**Figure 6:**
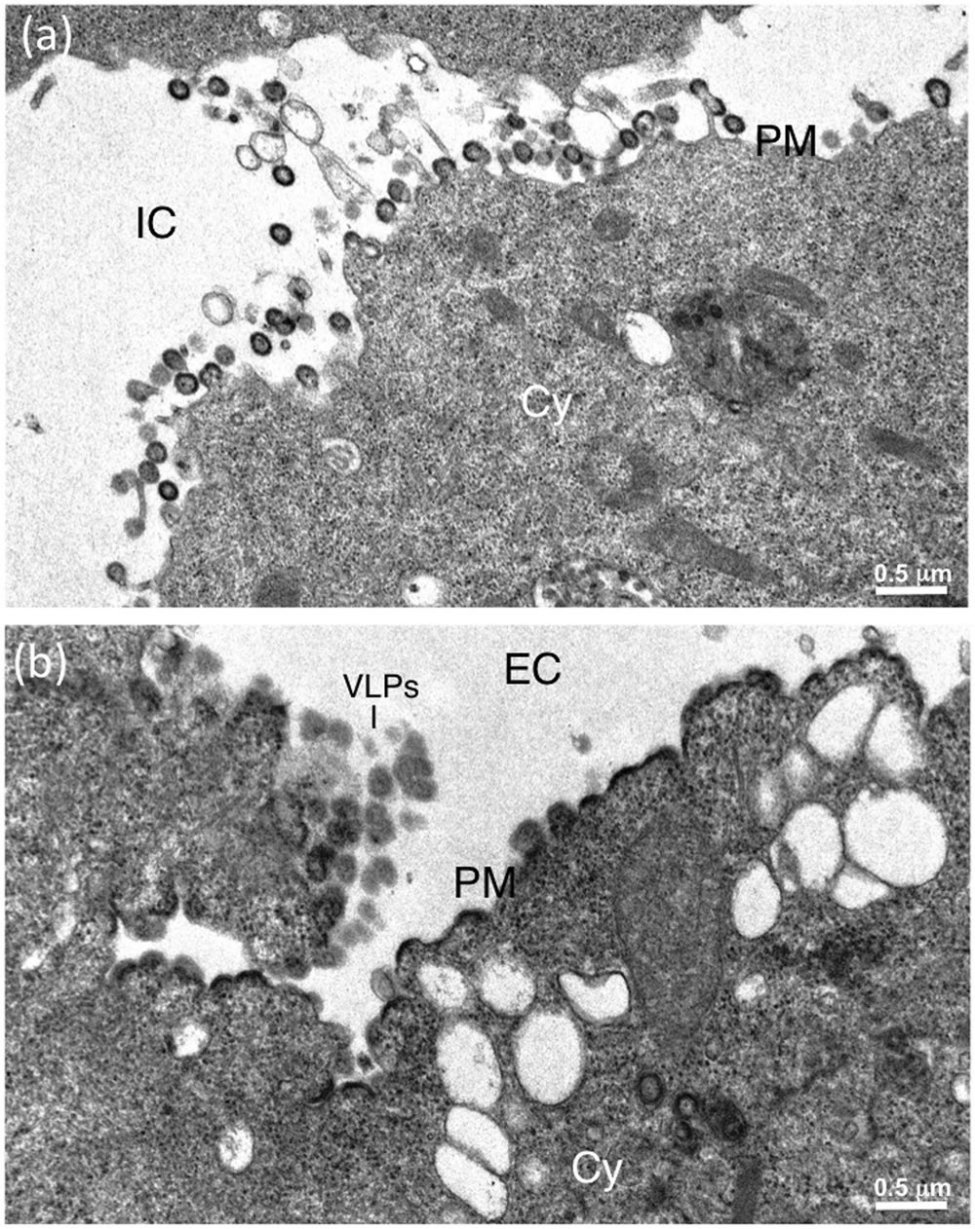
EM analysis of VLP assembly in αRep9A8-expressing insect cells. Sf/(Myr+)αRep9A8-GFP cells were infected with AcMNPV^*gag*^, harvested at 48hrs post-infection and processed for EM. The cells presented in panel **(a)** show VLP budding from the plasma membrane. The cell presented in panel **(b)** shows simultaneous VLP budding and abnormal accumulation of Pr55Gag polyprotein at the plasma membrane. IC, intercellular space; EC, extracellular space; PM, plasma membrane; Cy, cytoplasm.

**Figure 7:**
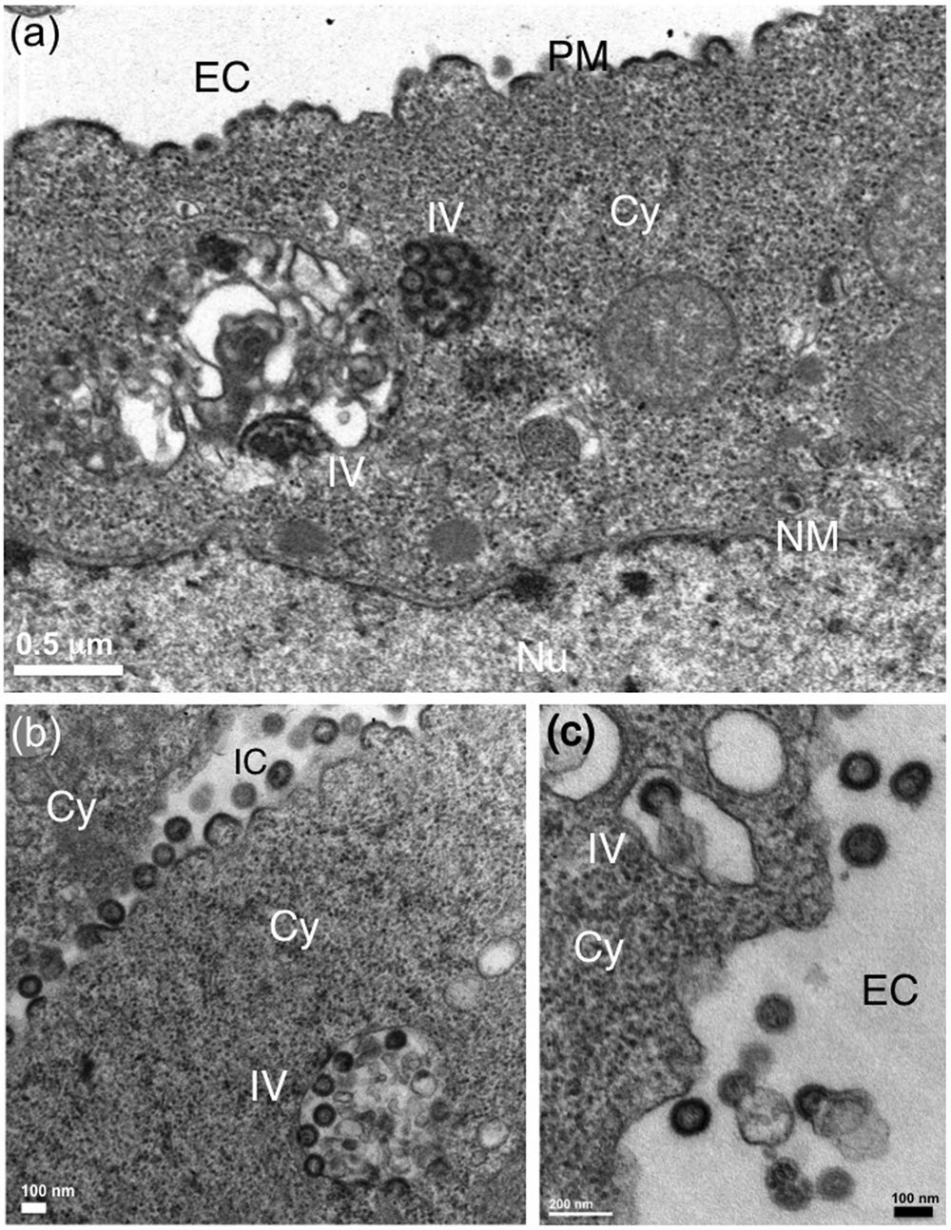
Intravesicular assembly of VLPs in αRep9A8-expressing insect cells. Electron micrographs of ultrathin sections of AcMNPV^*gag*^-infected Sf/(Myr+)αRep9A8-GFP cells at 48hrs post-infection. The cell presented in panel (a) shows the abnormal accumulation of Pr55Gag polyprotein at the plasma membrane, and the occurrence of VLPs within intracytoplasmic vesicles. Panels (b,c) present different cell sections at high magnification, showing simultaneous intravesicular budding and plasma membrane budding of VLPs. IC, intercellular space; EC, extracellular space; PM, plasma membrane; Cy, cytoplasm; IV, intracellular vesicle; Nu, nucleoplasm; NM, nuclear membrane.

The morphology of VLPs released from αRep-expressing cells were examined under the electron microscope (EM). From our previous studies^48,50,51^, membrane-enveloped VLPs released from the plasma membrane of AcMNPV^*gag*^-infected Sf9 cells appeared as quasi-spherical in shape, and homogeneous in size, with a diameter ranging from 110 to 130 nm (Fig. 4). By contrast, Sf/(Myr+)αRep4E3-GFP cells infected with AcMNPV^*gag*^ produced a majority of aberrant VLPs, irregular in size and shape, with the frequent occurrence of more than one single immature Gag protein core within the same membrane bud (Fig. 5). Likewise, Sf/(Myr+)αRep9A8-GFP cells infected with AcMNPV^*gag*^ showed aberrant VLPs (Fig. 6a), or arrested budding morphologies at their surface (Fig. 6b). Interestingly, a peculiar type of pattern was observed in (Myr+)αRep9A8-expressing cells, consisting of (i) the accumulation of αRep4E3 and αRep9A8 material at the inner leaflet of the plasma membrane (Figs 6b and 7a), and (ii) intravesicular budding of VLPs which seemed to be morphologically normal (Fig. 7a,b). This suggested that one of the consequences of the (Myr+)αRep9A8-mediated blockade of normal VLP budding at the plasma membrane was the redirection of Gag to the internal membranal compartment, and the budding of VLPs into intracytoplasmic vesicles.

Quantification of normal and aberrant VLPs released by control Sf9 cells exhibited less than 10% irregular particles (9.2%) out of a total number of 869 individual particles examined. However, Sf/(Myr+)αRep4E3-GFP cells released more than one third (35%) aberrant VLPs (*n* = 287), and Sf/(Myr+)αRep9A8-GFP cells about one quarter of the total (24.2%; *n* = 431) (see Supplementary Fig. S3).

Taken together, the results of these analyses suggested that both αRep4E3 and αRep9A8 negatively interfered with the assembly of Gag proteins into immature HIV-1 particles. These negative effects were not quantitative, as there was no significant difference in the yields of extracellular VLPs compared to control cells. The effects of αRep4E3 and αRep9A8 on Gag assembly were qualitative, as shown by the alteration of the structure of VLPs and the change in their cellular sites of assembly. In addition, EM analysis suggested two distinct phenotypes for αRep4E3 and αRep9A8.

### Co-encapsidation of Gag and N-myristoylated αRep proteins into VLPs

Since defective VLP assemblies were observed in the presence of αRep4E3 and αRep9A8, we hypothesized that these αRep proteins could be co-packaged with Gag precursor, resulting in altered VLP structure. To test this hypothesis, VLPs released from AcMNPV^*gag*^-infected Sf/(Myr+)αRep4E3-GFP and Sf/(Myr+)αRep9A8-GFP cells were purified by ultracentrifugation, and analyzed by SDS-PAGE and immunoblotting, using anti-Gag antibody to detect the Gag polyprotein, and histidine antibody to detect the His-tagged αRep-GFP proteins. An anti-histidine immunoreactive protein was detected in both types of VLP samples, at 59 and 55 kDa for αRep4E3-GFP and αRep9A8-GFP, respectively. These values corresponded to the respective molecular weights of the GFP-fused αRep proteins. The variations in the signal intensity of the 59-kDa and 55-kDa proteins throughout the gradient fractions followed the same pattern as that of the Pr55Gag band, with a maximum intensity in fractions of density ρ = 1.18 (see Supplementary Fig. S4). This suggested that N-myristoylated αRep4E3-GFP and αRep9A8-GFP proteins were able to interact with Pr55Gag at the VLP assembly sites, and to be copackaged with Pr55Gag into membrane-enveloped VLPs of aberrant structure. Of note, no αRep-GFP protein was detected at density 1.18 in culture media of Sf/(Myr+)αRep4E3-GFP and Sf/(Myr+)αRep9A8-GFP cells in the absence of the AcMNPV^*gag*^ vector (not shown).

### Antiviral activity of αRep4E3 and αRep9A8 proteins in human T-cells challenged with HIV-1

**Figure 8:**
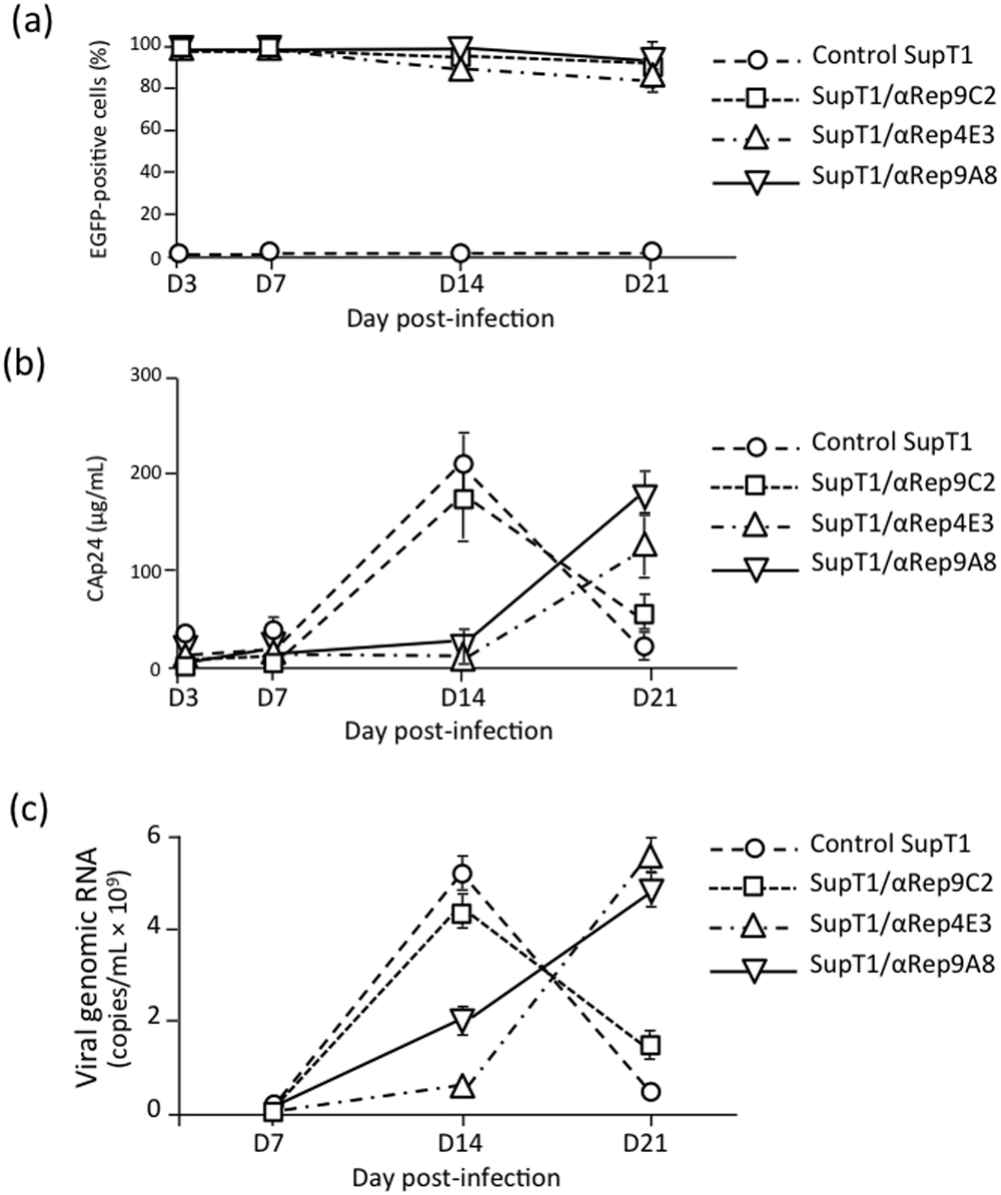
HIV-1 infection of αRep4E3- and αRep9A8-expressing SupT1 cells. (**a**) *Kinetics of GFP expression*. Control cells (unmodified SupT1 and SupT1/αRep9C2-GFP) and αRep-GFP-expressing SupT1 cells (SupT1/αRep4E3-GFP and SupT1/αRep9A8-GFP) were infected with HIV-1 at MOI 1, and monitored by flow cytometry from day 3 (D3) to D21 pi. Data presented are mean ± SD of triplicate experiments. (**b**) *Kinetics of* e*xtracellular release of viral particles*. Samples from culture media were collected at different time points pi, as indicated, and the levels of CAp24 antigen determined by ELISA. **(c)** *Kinetics of* e*xtracellular release of viral genomes*. Viral RNA yields were determined at D7, D14, and D21 pi in samples of culture media, and results expressed as copy number per mL.

The genes encoding αRep4E3-GFP, αRep9A8-GFP and αRep9C2-GFP (GFP-fused irrelevant αRep used as negative control) were transferred into SupT1 cells. Three αRep-expressing cell lines were successfully established, which were referred to as SupT1/αRep4E3-GFP, SupT1/αRep9A8-GFP and SupT1/αRep9C2-GFP cells, respectively (see Supplementary Fig. S5). Of note, the non-N-myristoylated versions of αRep proteins were expressed in SupT1 cells to allow them to diffuse and exert their possible antiviral activity in all cell compartments. The cells were infected with HIV-1 and the effect of the αRep proteins on HIV-1 infection was assessed by different methods, as described below. As shown by fluorescence microscopy and flow cytometry, the expression of αRep-GFP molecules was maintained at levels close to 100% throughout the time of the viral challenge (21 days; Fig. 8a).

#### Kinetics of HIV-1 particle production

CAp24-ELISA was used to evaluate the quantities of extracellular viral particles released from HIV-1-infected cells at days (D) 3, 7, 14, and 21 pi (Fig. 8b). In control cells, CAp24 was detected as early as at D7, with a peak at D14 followed by a decrease at D21 pi (Fig. 8b). However, CAp24 was barely detectable in samples from SupT1/αRep4E3-GFP and SupT1/αRep9A8-GFP cell cultures at D14 pi. The amounts detected were about 10-fold lower compared to that of control samples, and the CAp24 reached the levels observed in control D14-samples only at D21 pi. This indicated that in the cells expressing αRep4E3 and αRep9A8, there was a significant delay in the kinetics of HIV-1 replication and viral progeny production.

#### Extracellular release of HIV-1 genomes

The viral genome copies in the culture media followed the same kinetics as CAp24, with a similar delay observed in the presence of αRep4E3 and αRep9A8 (Fig. 8c). The viral load was maximal at D14 pi in control samples, and decreased at D21 due to cell apoptosis. In culture media of SupT1/αRep4E3-GFP and SupT1/αRep9A8-GFP cells, the virus loads slowly increased with time, and reached the maximal levels of control samples at D21 pi (Fig. 8c). A net difference between the αRep-induced inhibitory effects was observed at D14 pi, with a more pronounced negative effect with αRep4E3 (ca. 8-fold) compared to αRep9A8 (ca. 2-fold; Fig. 8c).

#### Cytopathic effects of HIV-1 on αRep-expressing SupT1 cells

**Figure 9:**
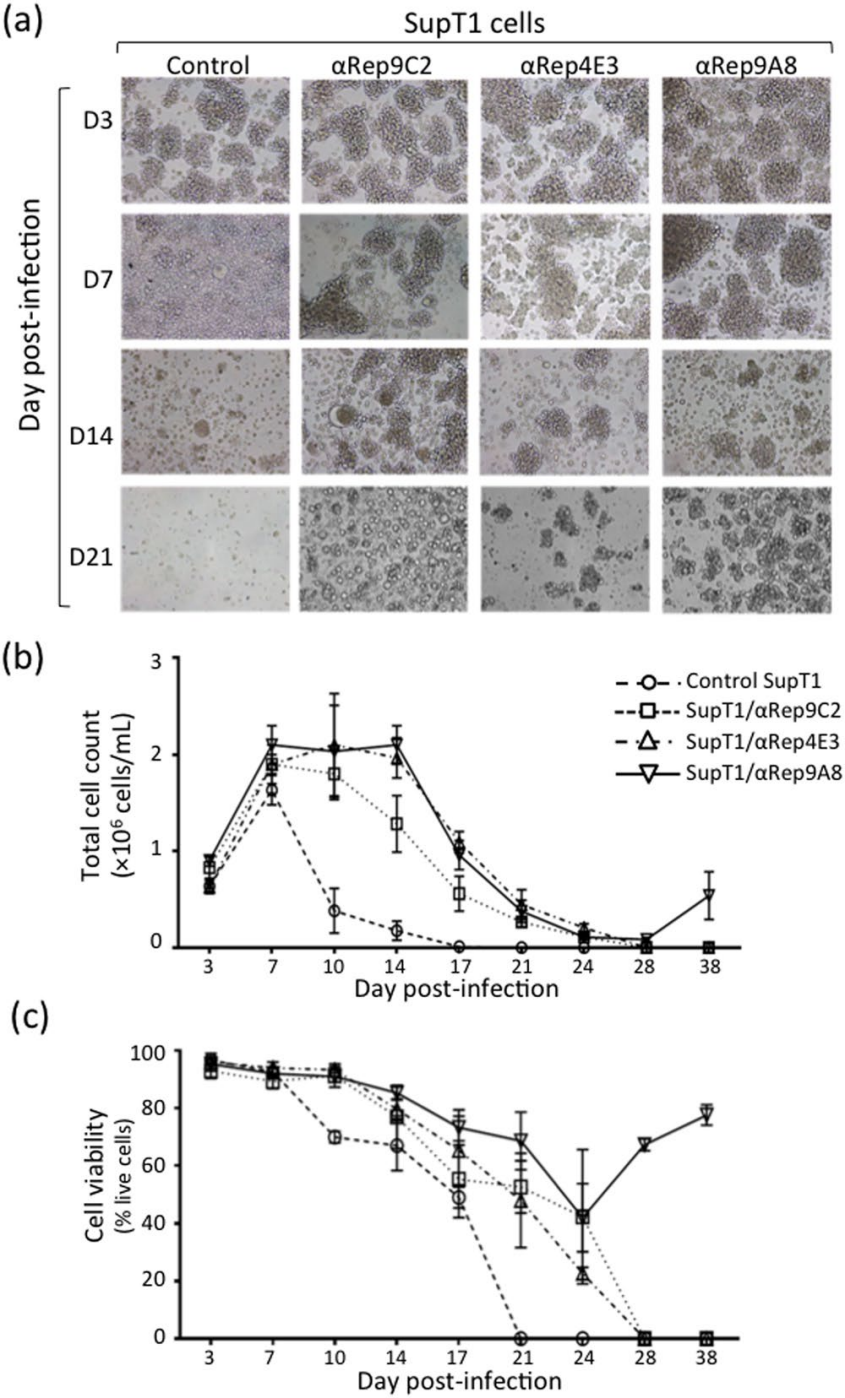
Cytopathogenicity of HIV-1 in αRep-expressing cells. HIV-1-infected cells were maintained in culture for several weeks. Control cell cultures consisted of unmodified SupT1 cells, and of SupT1/αRep9C2-GFP cells expressing an irrelevant αRep serving as negative control for αRep4E3 and αRep9A8. At different time intervals, as indicated, samples were collected and monitored for cell morphology, concentration, and viability. (**a**) Cell morphology, analyzed by phase contrast microscopy. (**b**) Total cell number per mL. (**c**) Percentage of viable cells. Bars represent the mean ± SD (*n* = 3).

HIV-1-infected SupT1 cells were maintained in culture for six weeks. They were monitored by phase microscopy for possible morphological alterations at different time points after infection (Fig. 9a). Formation of syncytia and premature cell death were clearly visible in control cell cultures, in which HIV-1 drastically reduced the proportion of live cells. These cellular alterations were delayed in SupT1/αRep4E3-GFP and SupT1/αRep9A8-GFP cultures (Fig. 9a). Cells samples were also collected at 3-day intervals, and controlled for cell count and cell viability. As expected, the cell concentration and viability decreased rapidly in control SupT1 cell cultures after D7 pi, and there was no more viable cells detected at D21 pi (Fig. 9b,c). The diminution in living cell numbers was delayed and more progressive in SupT1/αRep4E3-GFP and SupT1/αRep9A8-GFP cultures, with a decline starting only after D14 pi. A total cell loss was observed at D28 pi in SupT1/αRep4E3-GFP cultures, but a rebound in cell growth was observed after D28 pi in SupT1/αRep9A8-GFP cultures (Fig. 9b,c).

#### Proviral DNA status in HIV-1-infected aRep-expressing SupT1 cells

The viral genome integration in HIV-1-infected SupT1 cells, evaluated at D14 pi by quantitative PCR, showed that *Ct* values were not significantly different between control and αRep-expressing cells (see Supplementary Table S2). This suggested that resident, intracellular αRep4E3 and αRep9A8 did not prevent the viral genome integration in SupT1 cells, and that the antiviral effects of αRep4E3 and αRep9A8 proteins did not depend on viral integration.

### Molecular mechanisms of the αRep4E3- and αRep9A8-mediated antiviral effects

Two major parameters influence the infectivity of HIV-1: (i) the degree of proteolytic processing of Pr55Gag which controls the transition from immature to condensed, mature core; (ii) the ability of the virus to encapsidate its genomic RNA during its morphogenetic process, and thus the genome content of viral particles. It has been commonly considered that HIV-1, like most retroviruses, produce large numbers of defective particles lacking all or part of the viral genome, and that the infectivity index is usually in the low range of values, ≤1:1,000. However, the ratio of infectious to defective HIV-1 particles has been recently re-evaluated to 1:8^55^. Both Gag maturation and genome content were assayed in particles produced by αRep-expressing cells infected with HIV-1.

#### Proteolytic maturation of the Pr55Gag precursor

**Figure 10:**
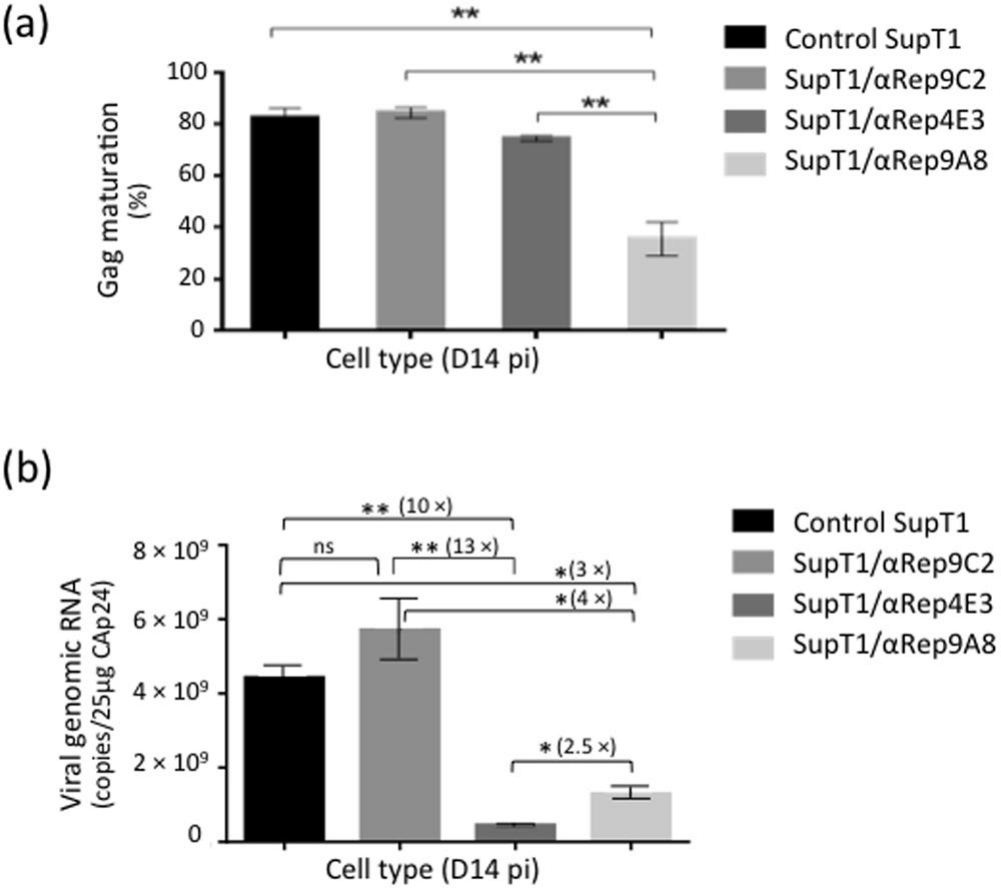
Antiviral activity of αRep4E3 and αRep9A8. (**a**), *PR-mediated maturation cleavage of Pr55Gag*. Control cells (unmodified SupT1 and SupT1/αRep9C2-GFP), and αRep-expressing cells (SupT1/αRep4E3-GFP and SupT1/αRep9A8-GFP) were infected with HIV-1 at the same MOI, and extracellular media collected. After normalization to the CAp24 level (80 µg per sample), the degree of proteolytic cleavage of Pr55Gag at the MA-CA junction by the viral protease (PR) was evaluated using an ELISA-based maturation assay. The bar graph represents the percentage of cleaved Pr55Gag (mean ± SD; *n = *3), estimated from the degree of accessibility and reactivity of a Gag-embedded MA epitope towards its specific monoclonal antibody, in competition with a free synthetic peptide reproducing its sequence (refer to *Materials & Methods*). **(b)**, *Viral genome packaging*. Bar graph representing the concentrations of HIV-1 genomes in culture media after normalization to CAp24 levels (genome copy number per 25 µg CAp24; mean ± SD; *n = 3*), and the fold changes in culture media of SupT1/αRep4E3-GFP and SupT1/αRep9A8-GFP cells, compared to control cell lines SupT1 and SupT1/αRep9C2-GFP. (**), *P* < 0.01; (*), *P* < 0.05; ns, not significant.

The viral protease-mediated processing of HIV-1 Pr55Gag is a sequential and high-order process. The initial cleavage occurs between SP1 and the NC domain, and the secondary (e.g. MA-CA) and tertiary (e.g. CAp24-SP1) cleavages occur at approximately 10-fold and 400-fold lower rates, respectively, than the initial cut^56,57^. The extent of Gag processing was assayed using HB-8975, a monoclonal antibody which recognized the C-terminal epitope DTGHSSQVSQNY only on free MAp17 protein, and not on intact Pr55Gag. After cleavage of the MA-CA junction, the exposed epitope can compete with a synthetic peptide of the same sequence for binding to HB-8975. The degree of competition in ELISA correlates with the extent of MA-CA cleavage, and provided a quantitative evaluation of the degree of Pr55Gag maturation, as shown in previous studies^58–60^. The results indicated that more than 85% viral particles which egressed from control, HIV-1-infected cells, and 75% from SupT1/αRep4E3-GFP cells, consisted of mature particles (Fig. 10a). This proportion was only 35% for SupT1/αRep9A8-GFP cells (Fig. 10a), suggesting that αRep9A8, but not αRep4E3, negatively interfered with the virus maturation.

#### Viral genome encapsidation

The efficiency of viral genome packaging in the different SupT1 cell lines was measured by the ratio of extracellular genome concentration to CAp24 concentration at D14 pi, and the results normalized to 25 µg CAp24 per sample, a value which corresponded to 5 × 10^7^ to 7 × 10^7^ infectious particles^61,62^, and 4 × 10^8^ to 6 × 10^8^ total particles^55^. The results showed that the RNA packaging function was altered in both SupT1/αRep4E3-GFP and SupT1/αRep9A8-GFP cell lines, but to different levels: a 10- to 13-fold reduction in RNA packaging efficiency was observed in αRep4E3-expressing cells, versus a 3- to 4-fold reduction in αRep9A8-expressing cells (Fig. 10b). This implied that the antiviral activity associated with αRep4E3 was predominantly directed against the viral genome packaging.

#### Infectivity of the viral progeny

**Figure 11:**
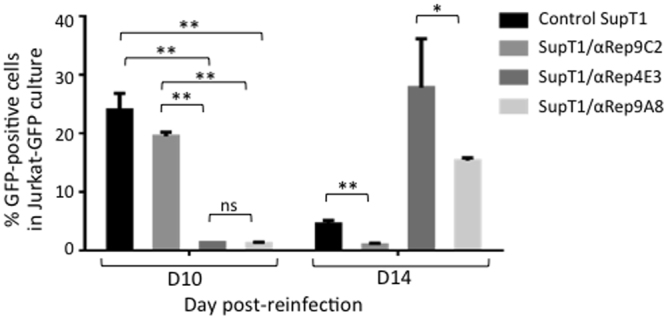
Infectivity of virus progeny released by HIV-1-infected, αRep-expressing SupT1 cells. Control cells (unmodified SupT1 and SupT1/αRep9C2-GFP), and αRep-expressing cells (SupT1/αRep4E3-GFP and SupT1/αRep9A8-GFP) were infected with HIV-1 at the same MOI. Samples from culture media were collected at D14 pi, and re-inoculated to Tat-dependent, indicator T-cells (Jurkat-GFP cells) after adjustment of the CAp24 levels to 15 µg per sample. Jurkat-GFP cells were harvested at D10 and D14 post-reinfection, and GFP-positive cells determined by flow cytometry. The results presented in the bar graph (mean ± SD; *n* = *3*) are the percentages of GFP-positive, fluorescent cells. (**), *P* < 0.01; ns, not significant.

The infectious titer of the viral progeny released from the different SupT1 cell lines was determined using Jurkat-GFP cells. This Jurkat T cell-based reporter cell line expresses CD4 and CXCR4 receptors, and enhanced GFP under the control of the HIV-1 LTR promoter, as a direct and quantitative marker of HIV-1 infection and Tat expression^63^. The results obtained at D10 post-reinfection (Fig. 11, leftmost bars) indicated that the infectious titers of viruses which egressed from SupT1/αRep4E3-GFP (1.4%) and SupT1/αRep9A8-GFP (1.25%) were significantly lower (15- to 17-fold), compared to that from the two control cell lines, SupT1 (24%) and SupT1/αRep9C2-GFP cells (19.5%). To evaluate the long-term effects of αRep proteins on virus titers, the reinfected Jurkat-GFP cells were maintained in culture, and analyzed by flow cytometry at D14 post-reinfection (Fig. 11, rightmost bars). The virus titers found in SupT1/αRep4E3-GFP samples were in the same range of values as those measured in control cells at D10. However, a significant difference (~2-fold) was observed between Jurkat-GFP cells reinfected with SupT1/αRep4E3-GFP (27.8%) and SupT1/αRep9A8-GFP supernatants (15.4%) at D14 post-reinfection. This indicated that αRep9A8 had a more pronounced negative effect on virus infectivity, compared to αRep4E3. Of note, the apparently low infectious titers of culture supernatants from control SupT1 cells at D14 post-reinfection were due to the massive cell death induced by HIV-1 in Jurkat-GFP cells at this time point, and no conclusion could be deduced from these values.

### Resident αRep9A8 protein in SupT1 cells conferred resistance to HIV-1 infection

**Figure 12:**
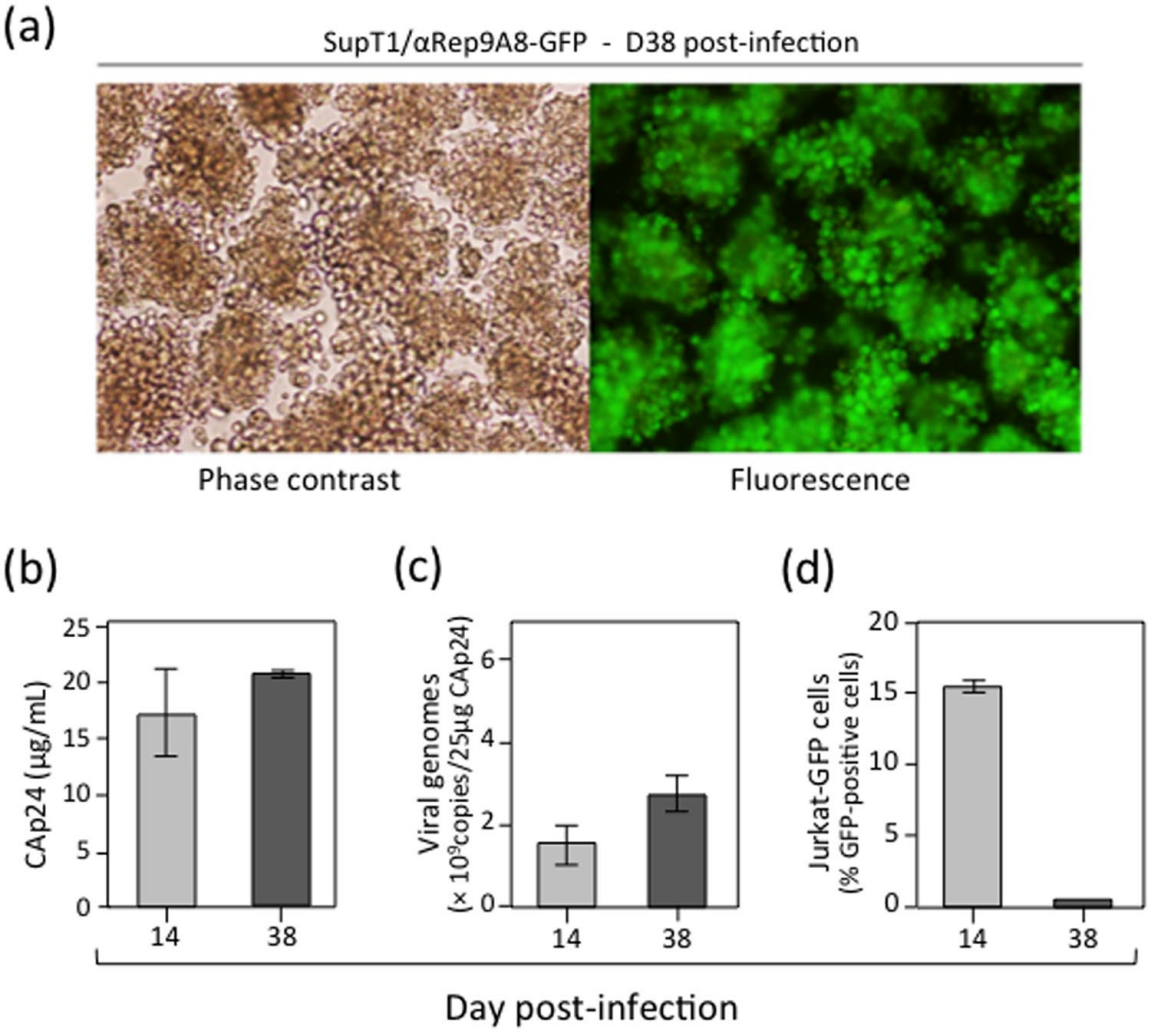
Resistance of SupT1/αRep9A8 cells to HIV-1 infection. (**a**), HIV-1-infected SupT1/αRep9A8-GFP cells were harvested at D38 pi, and cell morphology analyzed by light and fluorescence microscopy (magnification 100X). (**b**–**d)**, Samples from the cell cuture medium collected at D38 pi were assayed for (**b**) CAp24 levels, **(c)** viral RNA concentration, and **(d)** infectivity expressed as the percentage of GFP-positive Jurkat-GFP reporter cells. Data (mean ± SD; *n* = *3*) were compared with those obtained at D14 pi.

Cells which survived from the HIV-1-infected SupT1/αRep9A8-GFP cultures (refer to Fig. 9b,c) were collected at D38 pi, and were found to be healthy under the light and fluorescence microscopes (Fig. 12a). Samples from SupT1/αRep9A8-GFP culture supernatant at D38 pi were then assayed for extracellular virus yields. Viral particles and genomes were found in these samples, at concentrations almost equivalent to the values determined at D14 pi (Fig. 12b,c). Of note, these values were about 10-fold lower for CAp24 and 3-fold lower for the viral genome copies, compared to the corresponding parameters in control cell cultures at D14 pi (compare Fig. 12b and Fig. 8b, and Fig. 12c and Fig. 8c, respectively). The infectious virus titer of SupT1/αRep9A8-GFP culture supernatants at D38 pi was then determined using Jurkat-GFP indicator cells. The results showed that most of the extracellular HIV-1 particles released from SupT1/αRep9A8-GFP at this time pi were noninfectious (Fig. 12d).

## Discussion

In recent studies, we established the proof-of-concept that artificial proteins based on repeat motif scaffolds, could be used as intracellular antiviral agents, and we demonstrated their therapeutic potential as novel inhibitors of HIV-1^22,23,25^. In the present work, we applied this concept to another library of highly thermoresistant protein scaffolds, made up of alpha-helicoidal HEAT-like repeats, called αReps^26,27,29^. The bait used for the screening of our phage-displayed αRep library was the HIV-1 Pr55Gag-derived polypeptide CA_21_SP1-NC. It contained Gag determinants essential for achieving the late steps of the HIV-1 life cycle, i.e. the alpha-helical spacer peptide SP1 and its two flanking domains, the C-terminal domain of the CA protein and the full-length NC domain. Both SP1^30–35^ and NC^44,45^ have been used as targets for antiviral drugs against HIV-1.

**Figure 13:**
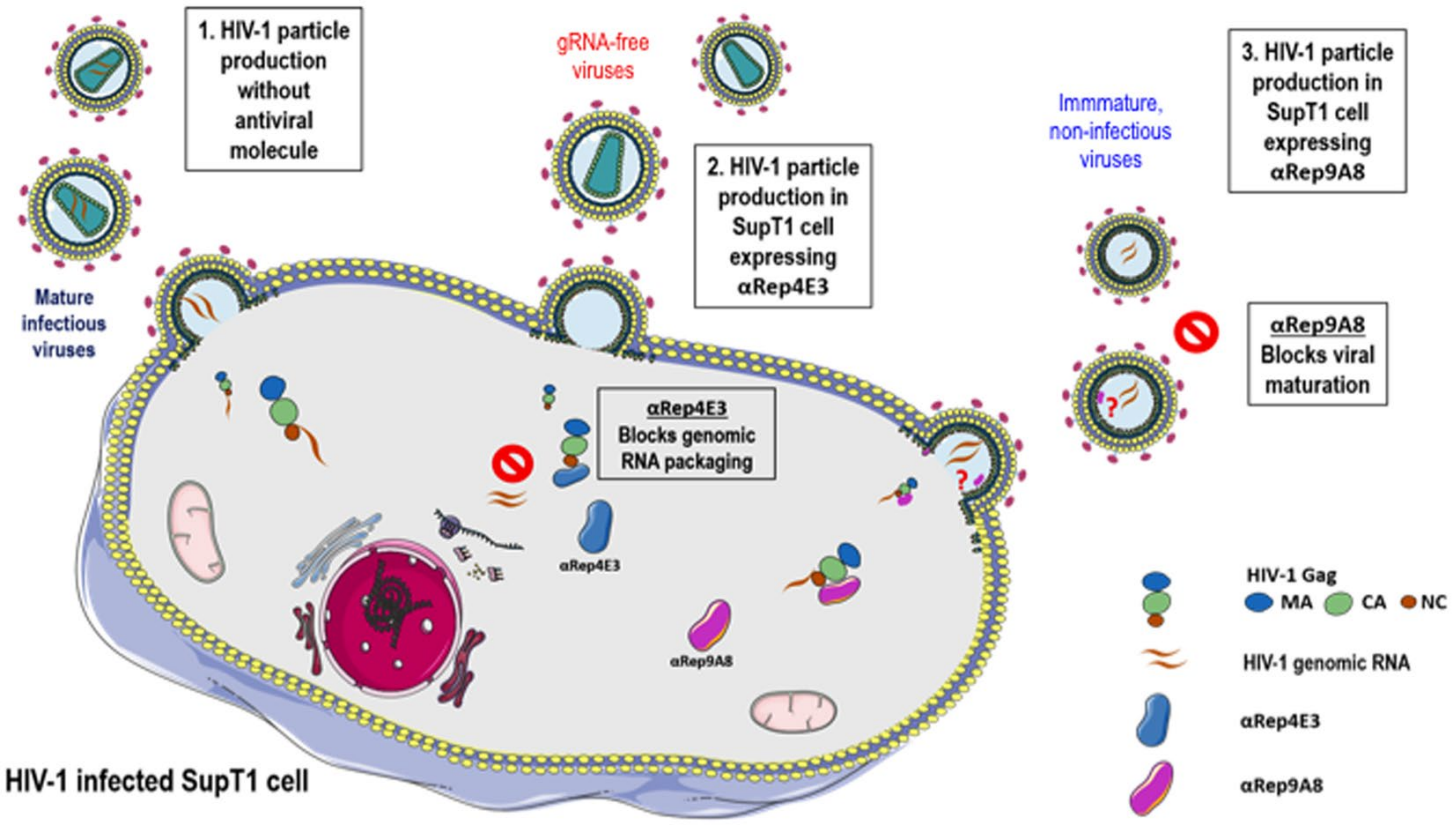
Schematic representation of the distinct antiviral effects of αRep4E3 and αRep9A8 in HIV-1-infected SupT1 cell. The normal pathway of HIV-1 assembly and egress is represented in (1); the antiviral effects of the αRep4E3 and αRep9A8 proteins are displayed in pathways (2) and (3). αRep4E3 (blue symbol) negatively interferes with Gag-RNA interaction and genome packaging; αRep9A8 (pink symbol) acts as a virus maturation inhibitor. Of note, the model presented in this figure was built by the first author, using a combination of elements available from the Servier Medical Art site http://smart.servier.com/. Servier Medical Art by Servier is licensed under a Creative Commons Attribution 3.0 Unported License (https://creativecommons.org/licenses/by/3.0/). As specified, users are free to share, copy and redistribute the material in any medium or format, and to adapt, remix, transform, and build upon the material for any purpose, even commercially.

Two αRep molecules, αRep4E3 and αRep9A8, with high binding activity to CA_21_SP1-NC were isolated from our screen. Both were found to exert a modest but significant inhibition on HIV-1, as shown by various biological assays. Their antiviral effects were independent of the proviral integration and occurred at the post-integration step. Differences were observed in their antiviral properties, as schematized in the model of Fig. 13. The αRep4E3 showed a marked negative effect on the viral genome packaging, while αRep9A8 interfered negatively with Gag maturation and its corollary, virus infectivity. More interesting, αRep9A8-expressing SupT1 cells acquired a long-term resistance to HIV-1, as shown by the absence of detectable infectious particles from the culture medium of αRep9A8-expressing SupT1 cells collected five weeks after HIV-1 challenge.

The mapping of the binding determinants of αRep4E3 and αRep9A8 on the viral target provided clues to their antiviral functions. The antiviral activity of αRep4E3, which mainly concerned the viral genome packaging, was consistent with the localization of its major binding site to the downstream zinc finger ZF2. The binding site of αRep9A8 spanned a wider NC domain, encompassing both ZF1 and ZF2 with a possible SP1 overlapping. The masking of SP1 cleavage sites would explain the negative interference of αRep9A8 with the PR-mediated Gag processing.

Since no binding of αRep9A8 to the GST-CA_21_-SP1 target was detected in our pull-down assays, it could be argued that the removal of the NC domain had altered the structure of SP1, and transformed its α-helical structure^64,65^ into a non-structured region. Even though this hypothesis cannot be totally excluded, it was not supported by the observation that isolated SP1 peptide in aqueous solution spontaneously folds into an α-helix without the aid of the NC, in a concentration-dependent manner. This α-helix forms at millimolar protein concentrations, i.e. at values equivalent to those found in virions^66–68^. Our results suggested that the negative interference of αRep9A8 with the viral PR resulted from the masking of the SP1 domain, indirectly *via* steric hindrance, rather than *via* direct binding.

The binding of αRep9A8 to a relatively large protein domain such as the NC raised a number of issues concerning the structure of the two partners. NMR studies of the three-dimensional structure of the HIV-1 NC protein have shown that the two ZF form a single folded protein domain characterized by spatial contacts between residues belonging to ZF1 and ZF2^69,70^. This globular structure is favoured by a highly conserved proline residue (P408 in HIV-1_LAI_ sequence) generating a bend in the linker separating the two ZF, and is stabilized by hydrophobic and aromatic interactions between residues belonging to ZF1 and ZF2^69,70^. The binding surface of an αRep with six internal repeats, such as αRep9A8, could be estimated to measure between 1,200 and 1,500 Å^2^, based on structural studies of αRep proteins co-crystallized with their target^26^. Such a surface area would theoretically be sufficient to interact with amino acid residues belonging to the two zinc fingers, and even to the adjacent SP1 linker.

The role of the NC and its two zinc fingers in the temporal control of reverse transcription, viral genome replication and packaging have been extensively documented, as well as their contribution to the structure of the viral core^36–40,71–73^. The observed antiviral effects of NC-interactors such as αRep4E3 and αRep9A8 might result from the blockage of one or several of these multiple functions. The application of real-time observation of fluorescent-tagged Gag in live cells^74^ to αRep-expressing live SupT1 cells should help us to dissect the kinetics and molecular mechanisms of the αRep-mediated antiviral effects, in particular their interference with Gag-RNA interaction and Gag assembly. NC also plays a role in the budding and release of HIV-1 particles by recruiting proteins of the cellular machinery necessary for viral budding, such as ALIX/AIP1^46,47^. Interestingly however, αRep4E3 and αRep9A8 showed no detectable negative effect on virus particle egress.

In terms of antiviral activity, the present study confirmed the feasibility of artificially produce antiviral restriction-like factors^22,23,25^. However, the immunogenenicity of such molecules will need to be assessed before envisaging any therapeutic applications. As a preliminary evaluation, we have performed the T-cell epitope prediction search for αRep4E3 and αRep9A8 (http://tools.iedb.org/processing/help/), and found negative MHC score values for both of them (unpublished data). The results of our comparative study of αRep4E3 and αRep9A8 properties suggested that, even though αRep4E3 was more detrimental to viral genome packaging compared to αRep9A8, the therapeutic advantage would be in favour of the virus maturation-interfering αRep9A8. In addition, the finding that αRep9A8 had the capability to block the production of infectious viral particles, and prolong the survival of HIV-1-infected cells, could significantly influence future strategies and choice of antivirals in anti-HIV-1 cell therapy.

## Methods

### Eukaryotic cells

#### Insect cells

*Spodoptera frugiperda (Sf9*) cells (Life Technologies) were maintained as monolayers at 28 °C in Grace’s insect medium (Life Technologies) supplemented with 15% fetal bovine serum, penicillin (100 Units/mL), and streptomycin (100 µg/mL). *Mammalian cells*. Human embryonic kidney cells (HEK293T), obtained from ATCC (Manassas, VA) were maintained in Dulbecco’s modified Eagle’s medium (DMEM; Life Technologies). Human T cells lymphoblastic lymphoma cells SupT1 and Jurkat LTR-GFP cells (JLTRG; obtained from the NIH AIDS Reagent Program) were grown in RPMI-1640 medium containing penicillin (100 U/mL), streptomycin (100 µg/mL), 2mM L-glutamine, and 10% FBS (HyClone). All cell lines were maintained in a humidified 37 °C incubator containing 5% CO_2_.

#### Bacterial cells and plasmid vectors

XL-1 Blue bacteria (Stratagene) were used as the host strain for generating the αRep phage library and for phage amplification. M15[pREP4] bacteria (Qiagen) were used to produce H_6_-tagged αRep proteins. *E. coli* BL21 was used as the host strain for producing GST-tagged proteins of HIV-1 Gag domains. The pQE-31 plasmid (Qiagen) was used for the production of H_6_-tagged recombinant αRep proteins. The pGEX-4T1 plasmid (GE Healthcare Life Sciences) was used to produce GST-tagged recombinant proteins at high levels.

### Viral vectors

#### Baculoviral vector

*Autographa california* multiple nuclear polyhedrosis virus (AcMNPV) has been used to construct the recombinant baculoviral vector AcMNPV^*gag*^ expressing the full-length, N-myristoylated wild-type HIV-1 Gag polyprotein, abbreviated Pr55Gag. The production of recombinant Pr55Gag and VLPs in AcMNPV^*gag*^-infected Sf9 cells have been descrived in detail in previous studies^48,50,51^.

#### VSVg-pseudotyped lentiviral vectors

CGW, a HIV-1-based self-inactivating (SIN) lentiviral vector of third-generation^25,75^ was used as the backbone vector to transfer the αRep genes into HEK293T and SupT1 cells. HEK293T cells at 70–80% confluence in 10-cm dish were co-transfected with the CGW transfer vector (10 µg/dish) carrying each one of the GFP-fused αRep genes, the packaging construct pMDLg/pRRE (6.5 µg), pRSV-Rev (2.5 µg), and pMD.2 G (3.5 µg). Lentiviral vector particles were harvested from the culture supernatant at 24 and 48 hrs post-cotransfection. High-titer vector stocks were obtained by ultracentrifugation through a 20% sucrose cushion. Viral titers were determined from the percentage of GFP-positive cells in HEK293T cultures infected with serial dilution of vector stock samples, at 48 hrs post-infection.

### HIV-1 stocks

Replication-competent HIV-1 NL_4-3_ virus, a X4-tropic strain of HIV-1, was produced by transient transfection of HEK293T cells by the pNL4-3 plasmid. In brief, monolayers of HEK293T cells at 4 × 10^6^ cells per 10-cm dish were transfected with 5 µg of the pNL_4-3_ plasmid using transIT-X2 transfection reagent (Mirus Bio, Madison, WI) according to the manufacturer’s protocol. The cells were allowed to grow for 48 hrs. HIV-1 virus particles in culture supernatant were then harvested by filtration through sterile syringe filters with a 0.45 µm pore size (Millex-HA filter unit; Merck Millipore, Hessen, Germany). HIV-1 stock was aliquoted and kept frozen at −80 °C. Virus particle titers were determined by conventional ELISA for CAp24-antigen, using the Genscreen ULTRA HIV Ag-Ab assay (Bio-Rad, Marnes-la-Coquette, France). Viral loads were determined using the COBAS® AmpliPrep/COBAS® TaqMan HIV-1 Test v2.0 (Roche Molecular Systems, Branchburg, NJ).

### Generation of HIV-1 target proteins

Our viral targets consisted of various glutathione-S-transferase (GST)-fused polyproteins derived from the HIV-1 GagPr55 precursor (LAI isolate). GST-CA_21_-SP1-NC included the 21 amino acids of the C-terminal domain of the capsid protein (CA), fused to the SP1 linker and the whole nucleocapsid (NC) domain, encompassing its two zinc fingers (ZF) until residue F433 (Fig. 1a). A panel of C-truncated mutants of GST-CA_21_SP1-NC were constructed by PCR amplification, using specific oligonucleotide primers (see Supplementary Table S1 **)**. The amplification products were purified by GeneJET PCR Purification kit, and a first step gene cloning was performed using InsTAclone PCR cloning kit (ThermoFisher Scientific, Waltham, MA). XL-1 Blue competent cells were transformed by the ligation products, and colonies were picked for PCR amplification, restriction enzyme analysis using *Bam*HI and *Xho*I, followed by DNA sequencing. Each construct was isolated from the pTZ57R/T vector and transferred to pGEX-GST expression vector using the same restriction sites, *Bam*HI and *Xho*I. The step of clonal identification was performed as previously described^21,22^.

Three pGEX-GST-*gag* plasmids were constructed: (i) pGEX-GST-CA_21_-SP1-NC; (ii) pGEX-GST-CA_21_-SP1-NCΔZF2; and (iii) pGEX-GST-CA_21_-SP1. BL21 cells were used for recombinant Gag protein production. BL-21 harboring each of the recombinant pGEX-GST-*gag* plasmids were cultured in 200 mL LB broth containing 100 µg/mL ampicillin and 1% (w/v) glucose, at 37 °C with shaking. When OD_600_ reached 0.8, protein expression was induced by addition of 0.1 mM isopropyl β-D-1-thiogalactopyranoside (IPTG), and maintained in culture at 30 °C overnight with shaking. Cells were pelleted by centrifugation at 1,200 × *g* for 30 min at 4 °C. Cell pellets were resuspended in PBS containing a cocktail of protease inhibitors (Roche Diagnostics GmbH), then lysed by sonication. Soluble proteins were purified by affinity chromatography on glutathione-agarose gel (ThermoFisher Scientific), following the protocol of the manufacturer, and analyzed by SDS-PAGE and Western blotting.

### Screening of the αRep library on the viral target

The construction of the αRep phage library 2.1 has been described in a previous study^27^. In brief, the αRep library was constructed by polymerization of synthetic microgenes corresponding to individual HEAT-like repeats, and the αRep proteins were expressed at the surface of M13-derived filamentous phages (phage display). In terms of diversity, our αRep library is estimated to contain 1.7 × 10^9^ independent clones.

For the αRep library screening, purified GST-CA_21_SP1-NC protein which represented our viral target was diluted at 5 µg/mL in coating buffer (TBST: 20 mM Tris/HCl, pH 8.0, 150 mM NaCl, 0.05% Tween-20) was immobilized on glutathione-coated microtiter ELISA plate (ThermoFisher Scientific) by incubation overnight at 4 °C in a moisture chamber. The coated wells were washed four times with TBST, and saturated with blocking solution (2% BSA in TBST; 200 µL/well) for 1 hr, after which an aliquot of the phage library was added to the GST-CA_21_SP1-NC coated wells, and incubated at room temperature (RT) for 1 hr with shaking. Several washes of the wells were then performed with TBST, and bound phages were eluted by three successive rounds of adsorption/elution. Phage elution was performed by acidic solution (0.1 M glycine-HCl buffer, pH 2.5) in the first two rounds. The last phage elution consisted of specific ligand elution^76,77^, by using a large excess of GST-CA_21_SP1-NC as the competitor. The population of αRep-displayed phages eluted from the GST-CA_21_SP1-NC bait was then amplified and subcloned in XL-1 Blue cells, as described for ankyrin scaffolds^21,22^.

Individual phage clones were selected and amplified as previously described^21,22^, and their respective binding activity towards the GST-CA_21_SP1-NC target was determined by ELISA. 100 µL-aliquots of purified GST-CA_21_SP1-NC (5 µg protein/mL) were diluted in PBS and dispensed into the wells of a glutathione-coated microtiter plate, then incubated overnight at 4 °C. The coated plate was washed four times with TBST, then blocked with TBST-BSA (200 µL per well) at RT for 1 hr with shaking. After a washing step, 100 µL-aliquots of each phage culture supernatant were added to the wells and incubated at RT for 1 hr, followed by HRP-conjugated mouse anti-M13 (GE Healthcare Life Sciences) diluted to 1:5,000 in TBST-BSA (100 µL-aliquot per well), and incubation proceeded at RT for an extra 1 hr. The wells were washed again, prior to the addition of 100 µL SureBlue™ TMB Microwell Substrate (KPL, Gaithersburg, MD). Reaction was stopped with 1 N HCl, and absorbance measured at 450 nm. Phage clones showing a high binding activity towards the immobilized target were sequenced and kept for cytoplasmic expression of individual αRep proteins.

### Expression and purification of αRep proteins

The *αRep* genes corresponding to strong binders to the GST-CA_21_-SP1-NC target were sub-cloned in pQE-31 plasmids, and used for transforming the bacterial strain M15[pREP4] strain, as previously described^21,22^. M15[pREP4] cells harboring the pQE31-αRep plasmids were grown at 37 °C in LB broth supplemented with ampicillin (100 µg/mL), kanamycin (25 µg/mL), and 1% (w:v) glucose with shaking. When absorbance at 600 nm reached 0.8, the αRep protein expression was induced by addition of 1 mM IPTG, and cells further incubated for 4 hrs at 30 °C with shaking. Bacteria were pelleted by centrifugation (1,200 × *g* for 30 min at 4 °C), and bacterial cell pellets resuspended in PBS containing a cocktail of protease inhibitors (Roche Diagnostics GmbH), then lysed by sonication. Bacterial cells lysates were clarified by centrifugation at 15,000 × *g* for 30 min at 4 °C. Soluble, H_6_-tagged recombinant αRep proteins were purified by affinity chromatography on HisTrap columns (GE Healthcare Life Sciences), and analyzed by SDS-PAGE and Western blotting.

### Protein pull-down assays

*E. coli* BL21 cells were transformed with plasmids pGEX-4T1 harboring the genes encoding the GST-CA_21_-SP1-NC, GST-CA_21_-SP1-NC∆ZF2, and GST-CA_21_-SP1 proteins, and bacterial cells cultured in Terrific broth (TR) medium supplemented with ampicillin (100 µg/mL), and glucose (1%; w/v) at 37 °C with shaking. When the OD_600_ of the cultures reached 0.8, protein expression was induced by addition of 0.1 mM IPTG, and the culture was further maintained at 30 °C for 16 hrs with shaking. Bacterial cells were then pelleted by centrifugation at 1,200 × *g* at 4 °C for 30 min, resuspended in PBS containing a cocktail of protease inhibitors (Roche Diagnostics GmbH), and sonicated. After clarification by centrifugation (15,000 × *g* at 4 °C, for 30 min), aliquots of supernatants containing GST-CA_21_-SP1-NC, GST-CA_21_-SP1-NC∆ZF2, or GST-CA_21_-SP1 were incubated with purified His-tagged αRep4E3 or αRep9A8 protein (300 µg) in PBS-0.01% Tween-20 (1 ml total volume), for 2 hrs at RT with shaking. Aliquots (20 µl) of glutathione-coated agarose beads (Pierce Glutathione Agarose; ThermoScientific, Waltham, MA) were then added to the reaction mixture, and further incubated for 1 hr at RT with shaking. Beads were centrifuged at low speed, washed 4 times with 0.05% Tween-20 in PBS, and resuspended and heat-denatured in SDS-PAGE loading buffer for Western blot analysis.

### SDS-PAGE and Western blotting

Samples normalized to equal amounts of total protein were separated by electrophoresis in SDS-containing 12%- or 15%-polyacrylamide gel. Gels were stained with PageBlue^TM^ protein staining solution (ThermoFisher Scientific), or used for Western blotting (WB). For WB, proteins separated on SDS-gels were electrically transferred to PVDF membrane (GE Healthcare, UK). After blocking with 5% skimmed milk in TBST, the PVDF membranes were incubated with various primary and secondary antibodies. GST-tagged proteins were detected using rabbit anti-GST antibody (Applied Biological Materials Inc., Richmond, Canada [ABM]). Gag proteins were detected using rabbit anti-Pr55Gag antibody (laboratory-made^31–33^). αRep proteins were detected using mouse monoclonal anti-H_6_ tag (ABM), in the conditions recommended by the manufacturer, or rabbit polyclonal anti-αRep protein (laboratory-made). Secondary antibodies consisted of HRP- or phosphatase-conjugated goat anti-mouse or anti-rabbit IgG antibody (KPL, Gaithersburg, MD), followed by specific chromogen reaction and color development. The protein bands on blots were scanned using the Photo Scan Lite application and the average relative band intensity values were obtained using Image Studio Lite Software Version 5.2.5.

### Construction of Sf9-derived cell lines stably expressing N-myristoylated αRep proteins

The integrative plasmid pIB/V5-His-TOPO vector (Life Technologies) was used for stable expression of GFP-tagged and N-myristoylated αRep proteins in Sf9 cells, under the control of the *OplE2* promoter. The sequence coding for a N-myristoylation signal^22,25^ was inserted at the 5′-end of the αRep-GFP genes to obtain N-myristoylated recombinant proteins, abbreviated (Myr+)αRep-GFP (Fig. 1c). Sf9 cells were transfected with the pIBV5-His-TOPO-based vectors (2.5 µg/10^6^ cells), using the DOTAP Liposomal Transfection Reagent (Roche Diagnostics GmbH). Transfected cells were maintained in complete Grace’s insect cell medium containing blasticidin (ThermoFisher Scientific) at 100 µg/mL. Two Sf9-derived cell lines were thus generated, referred to as Sf/(Myr+)4E3-GFP and Sf/(Myr+)9A8-GFP (see Supplementary Fig. S1). The expression of these proteins was monitored by fluorescence microscopy, and confirmed by SDS-PAGE and Western blot analysis using rabbit polyclonal anti-αRep antibody (laboratory-made).

### Isolation of extracellular HIV-1 virus-like particles (VLPs)

Sf9 cells were infected with AcMNPV^*gag*^ at an input multiplicity of 10 PFU/cell (MOI 10). Cells were harvested at 48 hrs postinfection (pi), and the culture supernatants were clarified by low-speed centrifugation. VLPs were recovered from the clarified supernatants using a two-step procedure comprising (i) a sucrose-step gradient centrifugation, followed by (ii) isopycnic ultracentrifugation^31–33,78^. In step (i), VLPs were pelleted through a sucrose cushion (20%, w:v, in TNE buffer; TNE: 100 mM NaCl, 10 mM Tris-HCl pH 7.4, 1 mM Na_2_EDTA) at 30 krpm for 1 hr at 15 °C in a Beckman SW55 rotor. For step (ii), pelleted VLPs were gently resuspended in 0.20–0.25 mL PBS, and centrifuged in a linear sucrose-D_2_O gradient. Linear gradients (10-mL total volume, 30–50%, w:v) were centrifuged for 18 hrs at 28 krpm in a Beckman SW41 rotor. The 50% sucrose solution was prepared in D_2_O buffered to pH 7.2 with NaOH, and the 30% sucrose solution was prepared in 10 mM Tris-HCl, pH 7.2, 150 mM NaCl, 5.7 mM Na_2_EDTA. Aliquots of 0.5 mL were collected from the top, and fractions analyzed for protein content by SDS-PAGE and immunoblotting, and by luciferase assays, as detailed below.

### Luciferase-based quantitative assay for HIV-1 Gag assembly and extracellular release of VLPs

The co-expression of the Luc-Vpr fusion protein by AcMNPV^LucVpr^ results in the incorporation of Luc-Vpr into Gag VLPs *via* the interaction of Vpr with the Gag p6 domain^33^. The levels of luciferase activity present in the pelletable fraction from the culture supernatant represented the amount of VLPs released. Aliquots (10^6^) of Sf9 (control cells), Sf/(Myr+)4E3-GFP and Sf/(Myr+)9A8-GFP cells were co-infected with two recombinant baculoviruses, AcMNPV^*gag*^ and AcMNPV^LucVpr^, at equal multiplicity of infection each (MOI 10). Co-infected cells were harvested at 48 hrs pi, and the amounts of extracellular VLPs released in the culture medium were quantitated using luciferase assays. VLPs were pelleted and lysed in KDT buffer (KDT: 0.1 M potassium phosphate buffer, pH 7.8, 1 mM DTT, containing 0.2% Triton × 100) for 30 min at 37 °C with vortexing every 10 min. The VLP-associated luciferase activity was measured using luciferin-ATP substrate (VivaGlo™ Luciferin, Promega) and the Lumat LB-9501 luminometer (Berthold Technologies, Bad Wildbad, Germany), as previously described^33^, and results expressed as relative light units (RLU) per μg protein. In order to correct for possible variations in the cellular expression of LucVpr, the values obtained with VLPs were normalized to the luciferase activity measured in the corresponding cell lysates, also expressed as RLU/μg protein.

### Electron microscopy

Control Sf9 cells, Sf/(Myr+)4E3-GFP and Sf/(Myr+)9A8-GFP cells were infected with AcMNPV^*gag*^ at MOI 10, harvested at 48 hrs post-infection, and processed for EM observation as described in previous studies^31–33,48^. Cells were fixed with 2.5% glutaraldehyde in 0.1 M phosphate buffer, pH 7.5, post-fixed with osmium tetroxide (2% in H_2_O) and treated with 0.5% tannic acid solution in H_2_O. The specimens were dehydrated and embedded in Epon (Epon-812; Fulham, Latham, NY). Ultrathin sections were stained with 2.6% alkaline lead citrate and 0.5% uranyl acetate in 50% ethanol, and poststained with 0.5% uranyl acetate solution in H_2_O. Grids were examined under a Jeol JEM-1400 electron microscope, equipped with an ORIUS™ digitalized camera (Gatan France, 78113-Grandchamp). For statistical EM analyses, a minimum of 50 grid squares containing 10 to 20 cell sections each were examined for counting VLPs budding at the cell surface, released in the external milieu or remaining in intracellular compartments^31–33,48^.

### Flow cytometry

The efficacy of cell transfection or transduction by lentiviral vectors was evaluated by flow cytometry. Cell samples were harvested at 48 hrs post-transfection or transduction. Cells were washed once with PBS, then with PBS containing 1% FBS-0.02% NaN_3_. After the washing steps, the cells were resuspended in 1% paraformaldehyde in PBS and analyzed by flow cytometry, using a BD Accuri^TM^C6 flow cytometer (BD Biosciences).

### Construction of SupT1 cell lines stably expressing αRep proteins

VSVg-pseudotyped CGW vectors harboring the genes encoding the non-N-myristoylated version of αRep4E3-GFP or αRep9A8-GFP proteins were produced in HEK293T cells. As negative control, an irrelevant, GFP-fused αRep (αRep9C2-GFP) was also inserted into CGW. Aliquots of SupT1 cells were transduced with VSVg-pseudotyped CGW-αRep-GFP vectors at MOI 1, using spinoculation at 2,500 × *g* and 32 °C for 2 hrs in growth medium containing Polybrene (8 µg/mL; Sigma-Aldrich, St. Louis, MO). At 16 hrs post-transduction, cells were washed 5 times with serum-free RPMI, and further incubated at 37 °C and 5% CO_2_ in fresh C-RPMI supplemented with FBS (10%), penicillin (100 U/mL) and streptomycin (100 µg/mL). The cells were then divided every 3 days and resuspended in fresh growth medium. Three stable cell lines, referred to as SupT1/αRep4E3-GFP, SupT1/αRep9A8-GFP and SupT1/αRep9C2-GFP cells, respectively, were isolated, and monitored by fluorescence microscopy and flow cytometry (BD Accuri^TM^ C6, BD Biosciences).

### Antiviral activity of αRep proteins in HIV-1-infected SupT1 cells

Control SupT1 cells and SupT1 cells stably expressing αRep4E3, αRep9A8 or αRep9C2 were maintained in growth medium for at least 4 weeks before incubation with HIV-1 NL_4-3_ virus inoculum at MOI 1 for 16 hrs. After rinsing with serum-free medium, the cells were resuspended in fresh growth medium, and maintained in culture for several weeks. HIV-1 particle yields were determined in the cell culture supernatants collected at 3-day intervals, using CAp24-ELISA (Bio-Rad, Marnes-la-Coquette, France), and viral genome copy numbers were assayed using the COBAS® AmpliPrep/COBAS® TaqMan HIV-1 Test v2.0 (Roche Molecular Systems, Branchburg, NJ).

The evaluation of viral genome integration could not be performed by using conventional *Alu-Gag* PCR assays on αRep-expressing SupT1 cells. These stable cell lines were generated by using the third-generation lentiviral vectors, which contain part of the U5 region required for the integration of the αRep gene into the cell chromosome. As a consequence, this region would give a positive signal in *Alu-Gag* PCR assays, even with noninfected cells. To avoid this drawback, viral genome integration was evaluated using SYBR green-based qRT-PCR assays performed on high molecular weight DNA isolated from infected cell lysates, with a pair of primers specific to an internal sequence of the *pol-prt* gene (see Supplementary Table S2). The kit used in these assays was the High Pure PCR Template Preparation Kit (Roche Product No. 11796828001), which has been developed to recover high molecular weight DNA from cellular genomic DNA, ranging from 30 to 50 kbp and free of contaminations with unintegrated viral cDNA.

The cell viability was evaluated using Trypan blue dye exclusion assays and the Countess™ Automated Cell Counter (Invitrogen, Thermo Fisher Scientific).

### ELISA-based HIV-1 Gag processing assay

The ELISA-based assay used to quantitate the extent of Gag processing relied on HB-8975, an anti-matrix (MA) monoclonal antibody issued from the hybridoma clone MHSVM33C9/ATCC HB-8975, and obtained from the American Type Culture Collection (ATCC, Manassas, VA). HB-8975 fails to react with its epitope (DTGHSSQVSQNY) located immediately upstream to the MA-CA junction when the MA domain is embedded in the Pr55Gag polyprotein precursor. After PR-mediated cleavage of the MA-CA junction, this epitope becomes fully exposed and accessible to HB-8975, and can compete with a free synthetic peptide mimicking its amino acid sequence. The degree of competition in ELISA correlates with the extent of MA-CA cut, and provides a quantitative evaluation of the degree of Pr55Gag maturation, as shown in previous studies^58–60^.

Microtiter plates were coated with 100 µL anti-MA HB-8975, diluted at 5 µg/mL in coating buffer (1 M NaHCO_3_ pH 9.6), and left overnight at 4 °C in a moisture chamber. Wells were washed 3 times with PBS-T, and incubated with 200 µL blocking solution (2% BSA in PBS) at RT for 1 hr to prevent non-specific binding. Next, 60 µL-aliquots of cell culture medium, normalized to the same CAp24 titer (80 µg/mL), were pre-incubated with Triton X-100 (1% final concentration) and 10 ng/mL biotinylated synthetic MAp17 C-terminal peptide at RT for 10 min. The mixtures were then added to the wells and incubation proceeded for 1hr. The binding reaction was revealed by incubation with HPR-conjugated streptavidin at RT for 1 hr, followed by addition of 100 µL-aliquots of TMB microwell peroxidase substrate. The reaction was stopped with 1 N HCl, and the optical density measured at 450 nm.

### Infectivity assays

Infectious titers were determined using JLTRG cells, a CD4/CXCR4-expressing Jurkat T cell-based reporter cell line, which harbors the enhanced *GFP* gene under the control of the HIV-1 LTR promoter. JLTRG cells (referred to as Jurkat-GFP cells in the present study) are Tat-dependent indicator T-cells which express GFP upon infection with HIV-1^63^. Samples from culture supernatants were adjusted to equal amounts of CAp24 (15 µg/mL) by dilution into fresh C-RPMI. Jurkat-GFP cells were seeded at 2.5 × 10^5^ cells/well in 96-well culture plate containing 8 µg/mL Polybrene (Sigma-Aldrich, St. Louis, MO). 100 μL-aliquots of the CAp24-normalized culture supernatants were added to the wells, and further incubated at 37 °C and 5% CO_2_ for 16 hrs. At 16 hrs pi, 100 μL-aliquots of fresh C-RPMI was added to the wells. Jurkat-GFP cells were harvested at D14 pi and assayed for GFP-positivity by flow cytometry^63,79–81^.

### Statistical analysis

Unless otherwise stated, results were expressed as mean ± SD of *n* experiments, using the GraphPad Prism version 6.01 for Windows (www.graphpad.com; GraphPad Software, La Jolla, CA). Differences were considered statistically significant when *P* < 0.05.

## Electronic supplementary material

Supplementary Figures & Tables
