# Supplementary material for: Alpha-helicoidal HEAT-like Repeat Proteins (αRep) Selected as Interactors of HIV-1 Nucleocapsid Negatively Interfere with Viral Genome Packaging and Virus Maturation
Source: Sci Rep. 2017 Nov 27;7:16335. doi: 10.1038/s41598-017-16451-w (PMC5703948; doi:10.1038/s41598-017-16451-w)
Supplement: Supplementary file 1 — Supplementary Figures & Tables [file 41598_2017_16451_MOESM1_ESM.pdf]

Alpha-helicoidal HEAT-like Repeat Proteins (αRep) Selected as Interactors of HIV-1 Nucleocapsid Negatively Interfere with Viral Genome Packaging and Virus Maturation

by

Sudarat Hadpech, Sawitree Nangola, Koollawat Chupradit, Kanda Fanhchaksai,
Wilhelm Furnon, Agathe Urvoas, Marie Valerio-Lepiniec, Philippe Minard,
Pierre Boulanger, Saw-See Hong and Chatchai Tayapiwatana

SUPPLEMENTARY INFORMATION

5 Figures

3 Tables

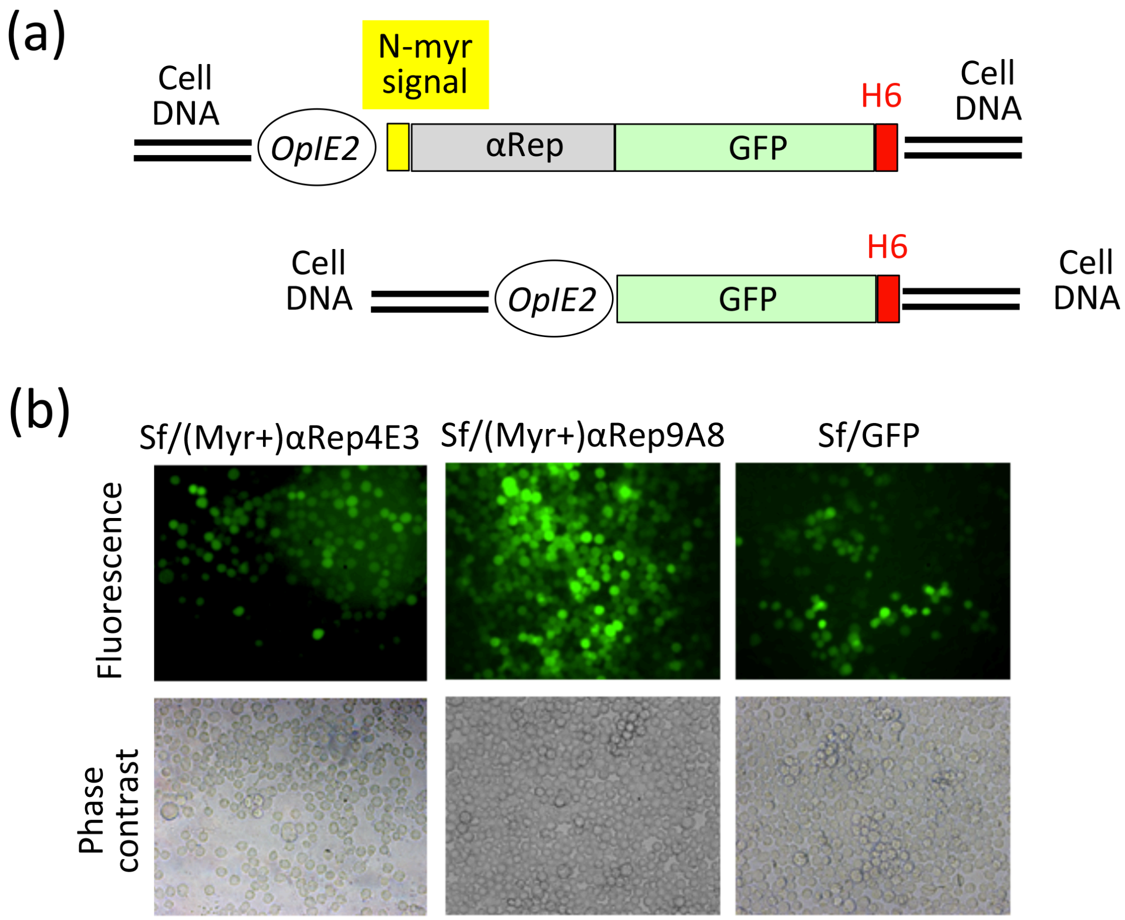

Supplementary Fig. S1. ***Generation of insect cells stably expressing αRep proteins***. Integrative plasmid pIB/V5-His was used for the construction of Sf9-derived, insect cell lines stably expressing GFP alone, or GFP-fused, N-myristoylated (Myr+) αRep molecules under the control of *OpIE2* promoter. **(a)**, Schematic gene constructs. **(b)**, Phase contrast and fluorescence microscopy of Sf/(Myr+)αRep4E3-GFP, Sf/(Myr+)αRep9A8-GFP and control Sf/GFP cells.

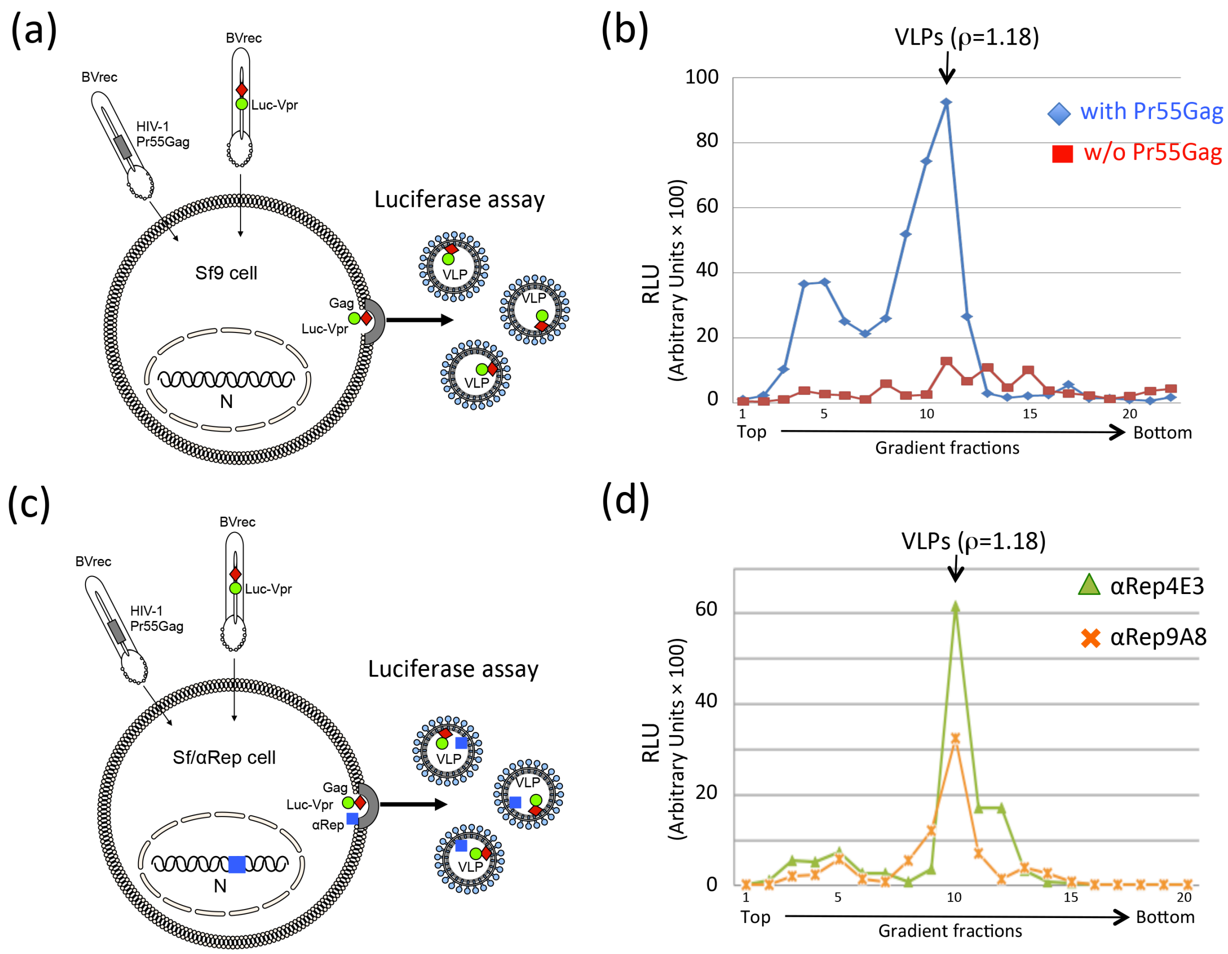

Supplementary Fig. S2. ***Luciferase-based assay of assembly and release of HIV-1 VLPs from recombinant baculovirus-infected insect cells*****. (a)**, Control, Sf9 cells infected with two recombinant baculoviruses (BVrec), AcMNPV^{gag} (expressing HIV-1 Pr55Gag) and AcMNPV$^{Luc\text{-}Vpr}$ (expressing the fusion protein Luciferase-Vpr; Luc-Vpr). Membrane-enveloped virus-like particles (VLPs) budding from the plasma membrane are constituted of Pr55Gag precursor, and contained Luc-Vpr fusion proteins coencapsidated with Pr55Gag, *via* Vpr-Gagp6 interaction. **(b)**, Luciferase activity associated with particulate material equilibrating at density 1.18 in isopycnic ultracentrifugation analysis (30%-50% sucrose-D_2O gradient). **(c)**, Sf/(Myr+)αRep4E3-GFP or (Myr+)αRep9A8-GFP cells infected with two recombinant baculoviruses, AcMNPV^{gag} and AcMNPV$^{Luc\text{-}Vpr}$. The αRep gene and protein are represented by blue squares; the Luc-Vpr fusion protein is represented by a green-and-red symbol. **(d)**, Luciferase assays show the incorporation of Vpr-fused luciferase into VLPs.

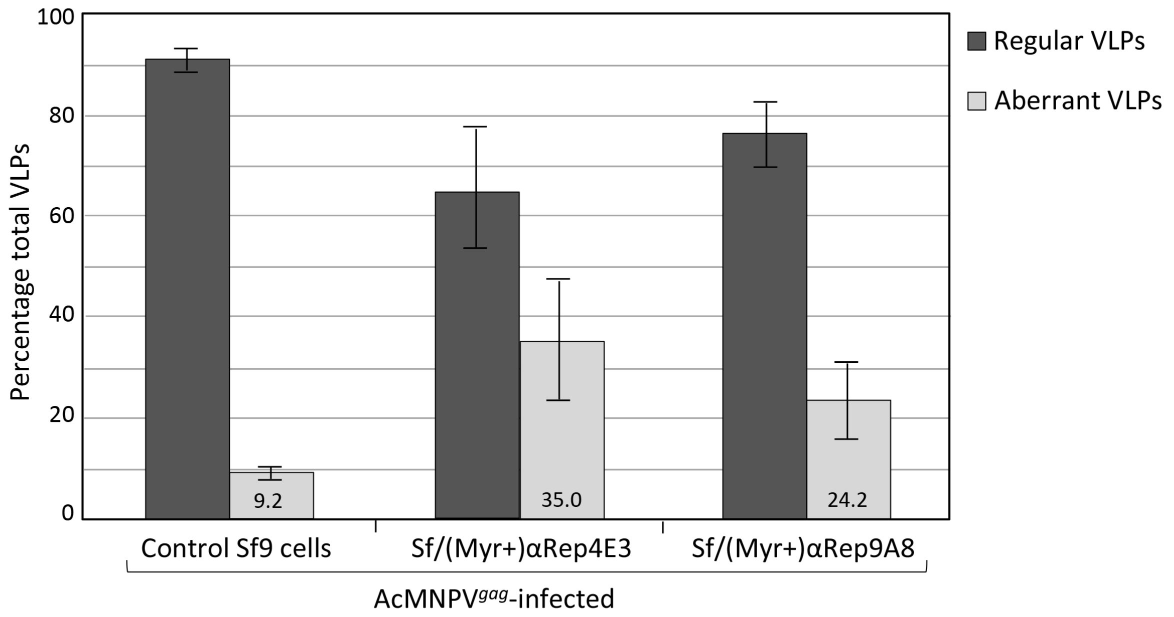

Supplementary Fig. S3. ***Proportion of regular versus aberrant VLPs released by αRep-expressing insect cells.*** AcMNPV^{gag}-infected control Sf9 cells, and Sf9-derived cells stably expressing (Myr+)αRep4E3-GFP or (Myr+)αRep9A8-GFP were harvested at 48 hrs pi and processed for electron microscopic analysis, as shown in Fig. 3 and Fig. 4. VLPs observed in more than 20 different ultrathin sections, were individually examined and counted. The bar graph represents the results of the counting, expressed as the percentage of the total VLPs counted in each cell sample (m ± SEM; n >200). The total numbers of VLPs counted in each specimen were n = 869 for control Sf9, n = 287 for Sf/(Myr+)αRep4E3-GFP, and n = 431 for Sf/(Myr+)αRep9A8-GFP cells.

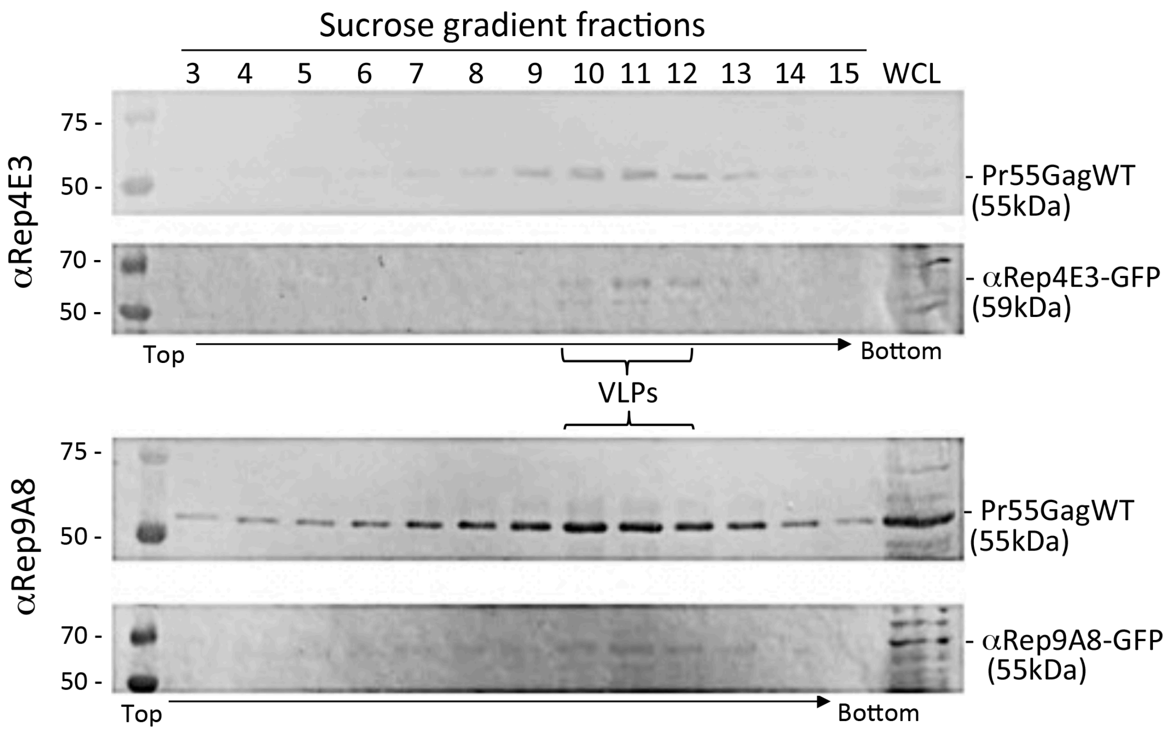

Supplementary Fig. S4. ***SDS-PAGE and Western blot analysis of extracellular VLPs from recombinant baculovirus-infected cells.*** Sf/(Myr+)αRep4E3-GFP (*top two panels*) and Sf/(Myr+)αRep9A8-GFP cells (*bottom two panels*) were infected with recombinant baculovirus AcMNPV^{gag}, and membrane-enveloped virus-like particles (VLPs) budding from the plasma membrane analyzed by isopycnic ultracentrifugation in a 30%-50% sucrose-D_2O gradient, as in Supplementary Fig. 2. The gradients fractions were analyzed by SDS-PAGE and immunoblotting, using anti-Gag and anti-His tag antibodies to detect Pr55Gag and αRep proteins, respectively. The fractions containing VLPs and corresponding to the relative density ρ = 1.18 are indicated by an accolade.

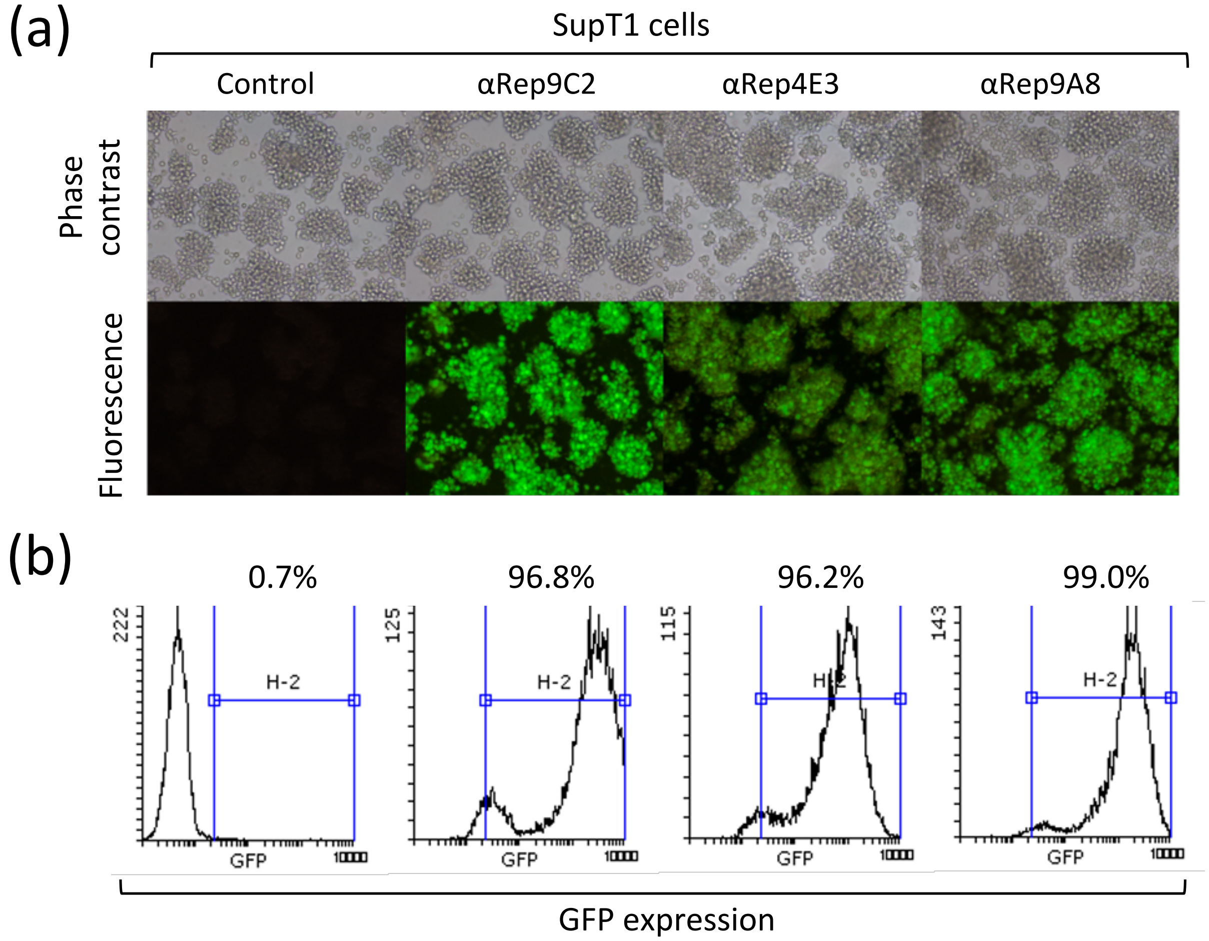

Supplementary Fig. S5. ***Monitoring of αRep-GFP expression in SupT1 cell lines***. SupT1 cells were transduced with a lentiviral vector carrying the genes coding for the αRep4E3-GFP or αRep4E3-GFP proteins. Control cells consisted of non-transduced SupT1 and SupT1 cells expressing the GFP-fused, irrelevant αRep protein αRep9C2-GFP. **(a)**, Cell morphology and GFP expression, monitored by phase contrast and fluorescence microscopy (magnification 100×). **(b)**, Flow cytometry analysis.

Supplementary Tables

Table S1. Primers used for the amplification of HIV-1 Gag constructs [(a)].

Gag constructs	Primers and sequences
Full-length viral target	
CA_{21}-SP1-NC	Fwd : 5'-GAT CCC TAG AAG AAA TGA TGA CA - 3'
	Rev: 5'-TCG AGT CAA TTA GCC TGT CTC TC - 3'
C-terminal deletion mutants	
	Fwd: 5’-GAG GAG GAG CTG GTT CCG CGT GGA TCC-3’
CA_{21}-SP1-NCΔZF2	Rev: 5’-GAG GAG GAG CTC GAG TCA CTT TTT CCT AGG-3’
CA_{21}-SP1	Rev: 5’-GAG GAG GAG CTC GAG TCA CAT TAT GGT AGC-3’

[(a)] Fwd, forward primer; Rev, reverse primer.

Table S2. HIV-1 proviral integration in SupT1 cells at D14 pi [(a)].

Sequence amplified [(b)]	Control SupT1	SupT1/αRep9C2	SupT1/αRep4E3	SupT1/αRep9A8
pol-prt gene	22.8 ± 0.2	21.4 ± 0.2	22.7 ± 0.4	23.6 ± 0.6

[(a)] Figures in the Table represent the Cts values, mean ± SD (*n=3*).

[(b)] The *pol-prt* gene sequence amplified spanned nucleotides 1,727 to 2,367 in HXB2 isolate.

Table S3. Primers *used for the amplification of the pol-prt gene sequence in proviral HIV-1.*

**Primers** [(a)]	**Sequences**
Fwd [(a)]	5'-TAA AAA AYT GGA TGA CAG AMA CCT T-3'
Rev	5'-TCA TTT TTG GTT TCC ATY TTC CTG G-3'

[(a)] Y and M were mixed bases, C and T for Y, A and C for M.
